# On the Generalized Decreasing Mean Time to Failure or Replaced Ordering

**DOI:** 10.1155/2021/7508812

**Published:** 2021-11-15

**Authors:** Haiyan Wang, Diantong Kang, Lei Yan

**Affiliations:** ^1^Business School, Zhejiang Wanli University, Ningbo, Zhejiang 315100, China; ^2^School of Mathematics and Statistics, Hexi University, Zhangye, Gansu 734000, China

## Abstract

In this paper, we establish two new stochastic orders, DMTFR (decreasing mean time to failure or replaced) and GDMTFR (generalized decreasing mean time to failure or replaced), and mainly investigate properties of the GDMTFR order. Some characterizations of the GDMTFR order are given. The implication relationships between the DMTFR and the GDMTFR orders are considered. Also, closure and reversed closure properties of the new order GDMTFR are investigated. Meanwhile, several illustrative examples that meet the GDMTFR order are shown as well.

## 1. Introduction and Preliminaries

In risk investment fields, we often need to compare or select two risk assets [1]. Similarly, two lifetimes of two systems or units also need to be compared in engineering technologies [2]. To do these things, some refined stochastic orders were defined in statistics. For more details on stochastic orders, one may refer to the studies by Maria Fernandez-Ponce et al. [3], Belzunce [4], Müller and Stoyan [5], Li and Yam [6], Shaked and Shanthikumar [7, 8], Zhao and Balakrishnan [9], Sunoj et al. [10], Kang [11, 12], Yan [13], Kang and Yan [14], Vineshkumar [15], and the references therein. However, sometimes, we need to compare two risk assets by the aid of the third referred system, such as when we compare two risk assets [16], their values are changeable with the settlement time or with the kind of valuation currency [17]. To solve this problem, we establish a new stochastic order by introducing a common measure factor, namely, referred function.

Let *X* be a nonnegative and absolutely continuous random variable. *X* has distribution function *F*_*X*_ with 0 being the left endpoint of its support, survival function ${\bar{F}}_{X}=1-F_{X}$, and density function *f*_*X*_. Let again *F*_*X*_^−1^ be the right-continuous inverse function of *F*_*X*_ defined by

(1) $$\begin{array}{c} F_{X}^{-1}\left(p\right)=\text{sup}\left\{x\;|\;F_{X}\left(x\right)\leq p\right\},\;0<p<1. \end{array}$$

In many important real areas, such as reliability, economics, management sciences, information sciences, and other related fields, stochastic comparisons are paid much attention and have been used as sharp tools in dealing with some random problems in recent years. Based on comparisons of residual life, mean residual life, mean inactivity time, failure rate, and many aging concepts are presented earlier or later. In these aspects, interested readers can refer to the studies by Barlow and Proschan [18], Shaked et al. [7], Shaked and Shanthikumar [8], Müller and Stoyan [5], Kochar et al. [19], Ahmad et al. [20], Knopik [21], Kochar et al. [19], and Li and Shaked [22] for some existing results.

In reality, a nonnegative random variable *X* is often called a life representing the lifetimes of a device. The life distribution classes IFR (increasing failure rate), IFRA (increasing failure rate average), DMRL (decreasing mean residual life), NBUE (new better than used in expectation), and MTFR (mean time to failure or replaced) classes of life distributions are commonly used to describe the ageing features of units or systems, for example, see Barlow and Proschan [18].

In particular, it is well known that (see Barlow and Campo [23], Klefsjö [24], Marschall [25])

(2) $$\begin{array}{c} \text{IFR}\subset \text{DMRL}\subset \text{NBUE}. \end{array}$$

Knopik [21] introduced a new ageing class MTFR such that

(3) $$\begin{array}{c} \text{IFR}\subset \text{MTFR}\subset \text{NBUE}. \end{array}$$

It is worthwhile to mention that, in Barlow's study [26], it is proved for the absolutely continuous distribution that

(4) $$\begin{array}{c} \text{IFRA}\subset \text{MTFR}. \end{array}$$

Kochar et al. [19] showed that, for any random variable, the inclusion (4) is also valid.

Consider an age replacement police as the one in which a unit is replaced by *t* time units after installation or at failure, whichever occurs first, and then the expected value for the first time to an in-service is (see Barlow and Proschan [18])

(5) $$\begin{array}{c} M_{X}\left(t\right)=\frac{\int_{0}^{t}{\bar{F}}_{X}\left(x\right)\text{d}x}{F_{X}\left(t\right)}\text{, for }t\in \left\{x:F_{X}\left(x\right)>0\right\}. \end{array}$$

When *M*_*X*_(*t*) is monotonic, the case was considered by Barlow and Campo [23], Marschall and Proschan [27], and Klefsjö [24]. In Knopik [21], and the ageing class MTFR (mean time to failure or replaced) of lifetime distribution is introduced, and it is proved that the MTFR class is closed under the operation of maximum for independence. In Kochar et al. [19], it is showed that the class MTFR is closed under weak convergence of distributions and convolution, and the dual family MTFR^*D*^ is closed under noncrossing mixtures.

**Definition 1 .**

The random variable *X* belongs to the mean time to failure with replacement (MTFR) class if the function *X* is decreasing for *t* ∈ {*x|F*_*X*_(*x*) > 0}.

Assume that *X* has finite mean *𝔼*(*X*)=*μ*_*X*_. The residual life of *X* at time *t* > 0 is defined as *X*_*t*_=[*X* − *t∣X* > *t*]. Then, the mean residual life of *X* at time *t* > 0 is

(6) $$\begin{array}{c} \mu_{X}\left(t\right)=E\left(X_{t}\right)=\frac{\int_{t}^{+\infty }{\bar{F}}_{X}\left(x\right)\text{d}x}{{\bar{F}}_{X}\left(t\right)},\;\text{for }t\geq 0. \end{array}$$

If *μ*_*X*_(*t*) is decreasing, then we say that *X* (or *F*_*X*_) is in the decreasing mean residual (DMRL) life distribution class and denoted by *X*(or *F*_*X*_) ∈ DMRL.

Emad-Edlin [28] proposed and studied the decreasing mean residual life (DMRL) ordering. Maria Fernandez-Ponce et al. [3] and Belzunce [4] proposed and studied the right spread order. Subsequently, Kochar et al. [19] proposed and studied the total time on test transform order and excess wealth order which is equivalent to the right spread order.

The total time on test (TTT) transform functions of *X* and *Y* are defined, respectively, as

(7) $$\begin{array}{c} T_{X}\left(p\right)\equiv \int_{0}^{F_{X}^{-1}\left(p\right)}{\bar{F}}_{X}\left(x\right)\text{d}x,\\ T_{Y}\left(p\right)\equiv \int_{0}^{G_{Y}^{-1}\left(p\right)}{\bar{G}}_{Y}\left(x\right)\text{d}x,\;\text{for all }p\in \left(0,1\right). \end{array}$$

The right spread (RS, for short) functions of *X* and *Y* are defined as, for all *p* ∈ (0,1), respectively,

(8) $$\begin{array}{c} S_{X}\left(p\right)=\int_{F_{X}^{-1}\left(p\right)}^{\infty }{\bar{F}}_{X}\left(x\right)\text{d}x=E\left[{\left(X-F_{X}^{-1}\left(p\right)\right)}_{+}\right]=E\left[\max X-F_{X}^{-1}\left(p\right),0\right],\\ S_{Y}\left(p\right)=\int_{G_{Y}^{-1}\left(p\right)}^{\infty }{\bar{G}}_{Y}\left(x\right)\text{d}x=E\left[{\left(Y-G_{Y}^{-1}\left(p\right)\right)}_{+}\right]=E\left[\max\left\{Y-G_{Y}^{-1}\left(p\right),0\right\}\right], \end{array}$$

where ${\bar{F}}_{X}=1-F_{X}$ and ${\bar{G}}_{Y}=1-G_{Y}$ are the survival functions of *F*_*X*_ and *G*_*Y*_, respectively, and (*x*)_+_=max{*x*, 0}.

Now, we recall several stochastic orders from Shaked and Shanthikumar [8].

**Definition 2 .**

Let *X* and *Y* be two nonnegative random variables with distribution functions *F*_*X*_, *G*_*Y*_ such that *F*_*X*_(0)=*G*_*Y*_(0)=0, survival functions ${\bar{F}}_{X}\equiv 1-F_{X},{\bar{G}}_{Y}\equiv 1-G_{Y}$, right-continuous inverse functions *F*_*X*_^−1^, *G*_*Y*_^−1^, and finite means *μ*_*X*_, *μ*_*Y*_, respectively [8].

(1) *X* is said to be smaller than *Y* in the TTT transform order (denoted by *X*≤_ttt_*Y*) if
  (9) $$\begin{array}{c} \int_{0}^{F_{X}^{-1}\left(p\right)}{\bar{F}}_{X}\left(x\right)\text{d}x\leq \int_{0}^{G_{Y}^{-1}\left(p\right)}{\bar{G}}_{Y}\left(x\right)\text{d}x,\;\text{for all }p\in \left(0,1\right). \end{array}$$
(2) *X* is said to be smaller than *Y* in the right spread order (denoted by *X*≤_rs_*Y*) if
  (10) $$\begin{array}{c} \int_{F_{X}^{-1}\left(p\right)}^{\infty }{\bar{F}}_{X}\left(x\right)\text{d}x\leq \int_{G_{Y}^{-1}\left(p\right)}^{\infty }{\bar{G}}_{Y}\left(x\right)\text{d}x,\;\text{for all }p\in \left(0,1\right). \end{array}$$
(3) *X* is said to be smaller than *Y* in the NBUE (new better than used in expectation) order (denoted by *X*≤_nbue_*Y*) if
  (11) $$\begin{array}{c} \frac{\int_{F_{X}^{-1}\left(p\right)}^{\infty }{\bar{F}}_{X}\left(x\right)\text{d}x}{\int_{G_{Y}^{-1}\left(p\right)}^{\infty }{\bar{G}}_{Y}\left(x\right)\text{d}x}\leq \frac{\mu_{X}}{\mu_{Y}},\;\text{for all }p\in \left(0,1\right). \end{array}$$
(4) *X* is said to be smaller than *Y* in the location independent riskier order (denoted by *X*≤_lir_*Y*) if
  (12) $$\begin{array}{c} \int_{0}^{F_{X}^{-1}\left(p\right)}F_{X}\left(x\right)\text{d}x\leq \int_{0}^{G_{Y}^{-1}\left(p\right)}G_{Y}\left(x\right)\text{d}x,\;\text{for all }p\in \left(0,1\right). \end{array}$$
(5) *X* is said to be smaller than *Y* in the DMRL order (denoted by *X*≤_dmrl_*Y*) if
  (13) $$\begin{array}{c} \frac{\mu_{Y}\left[G_{Y}^{-1}\left(p\right)\right]}{\mu_{X}\left[F_{X}^{-1}\left(p\right)\right]}=\frac{\int_{G_{Y}^{-1}\left(p\right)}^{\infty }{\bar{G}}_{Y}\left(x\right)\text{d}x}{\int_{F_{X}^{-1}\left(p\right)}^{\infty }{\bar{F}}_{X}\left(x\right)\text{d}x} \end{array}$$
is increasing in *p* ∈ (0,1).

From Definition 1, we easily have the following result without proof.

**Lemma 1 .**

Let *X* and *Y* be two continuous and nonnegative random variables with respective distribution functions *F*_*X*_ and *G*_*Y*_, density functions *f*_*X*_ and *g*_*Y*_, and the right-continuous inverse functions *F*_*X*_^−1^(*p*) and *G*_*Y*_^−1^(*p*) of *F*_*X*_ and *G*_*Y*_, respectively. Then, the following statements are equivalent:

(1) *X*≤_ttt_*Y*.
(2) The inequality
  (14) $$\begin{array}{c} \int_{0}^{F_{X}^{-1}\left(p\right)}{\bar{F}}_{X}\left(x\right)\text{d}x\leq \int_{0}^{G_{Y}^{-1}\left(p\right)}{\bar{G}}_{Y}\left(x\right)\text{d}x,\;\text{for all }p\in \left(0,1\right), \end{array}$$
is valid.
(3) The inequality
  (15) $$\begin{array}{c} \int_{0}^{t}{\bar{F}}_{X}\left(x\right)\left[\frac{f_{X}\left(x\right)}{g_{Y}\left[G_{Y}^{-1}\left(F_{X}\left(x\right)\right)\right]}-1\right]\text{d}x\geq 0,\;\text{for all }t>0, \end{array}$$
is valid.

**Definition 3 .**

Let *X* and *Y* be two nonnegative random variables with distribution functions *F*_*X*_, *G*_*Y*_ such that *F*_*X*_(0)=*G*_*Y*_(0)=0, survival functions ${\bar{F}}_{X}\equiv 1-F_{X},{\bar{G}}_{Y}\equiv 1-G_{Y}$, density functions *f*_*X*_, *g*_*Y*_, and right-continuous inverse functions *F*_*X*_^−1^, *G*_*Y*_^−1^, respectively.

1. *X* is said to be smaller than *Y* in the usual stochastic order (denoted by *X*≤_st_*Y*), if ${\bar{F}}_{X}\left(x\right)\leq {\bar{G}}_{Y}\left(x\right)$ for all *x* > 0.
2. *X* is said to be smaller than *Y* in the star-shaped order (denoted by *X*≤_*∗*_*Y*), if the function *G*_*Y*_^−1^(*F*_*X*_(*x*))/*x* is increasing in *x* > 0.
3. *X* is said to be smaller than *Y* in the convex order (denoted by *X*≤_c_*Y*), if the function *G*_*Y*_^−1^(*F*_*X*_(*x*)) is convex for *x* > 0.
4. *X* is said to be smaller than *Y* in the dispersive order (denoted by *X*≤_disp_*Y*), if *f*_*X*_[*F*_*X*_^−1^(*p*)] ≥ *g*_*Y*_[*G*_*Y*_^−1^(*p*)] for all *p* ∈ (0,1).

Let *X* and *X* be two absolutely continuous nonnegative random variables with respective distribution functions *F*_*X*_ and *G*_*Y*_ such that *F*_*X*_(0)=*G*_*Y*_(0)=0, and density functions *f*_*X*_ and *g*_*Y*_, and right-continuous inverse functions of *F*_*X*_^−1^ and *G*_*Y*_^−1^, respectively.

We say that *X* is smaller than *Y* in the increasing concave order (denoted by *X*≤_icv_*Y*) if

(16) $$\begin{array}{c} \int_{0}^{t}{\bar{F}}_{X}\left(x\right)\text{d}x\leq \int_{0}^{t}{\bar{G}}_{Y}\left(x\right)\text{d}x,\;\text{for all }t>0. \end{array}$$

Here,

(17) $$\begin{array}{c} \int_{0}^{t}{\bar{F}}_{X}\left(x\right)\text{d}x=E\left[\min\left\{X,t\right\}\right],\\ \int_{0}^{t}{\bar{G}}_{Y}\left(x\right)\text{d}x=E\left[\min\left\{Y,t\right\}\right]. \end{array}$$

Hence, the order ≤ _icv_ is a direct comparison for the mean service times of *X* and *Y*. In insurance theory, $\int_{0}^{d}{\bar{F}}_{X}\left(x\right)\text{d}x=𝔼\left[\min\left\{X,d\right\}\right]$ is the average losses undertaken by the insured when the deductible excess is *d*. Hence,

(18) $$\begin{array}{c} T_{X}\left(p\right)\equiv \int_{0}^{F_{X}^{-1}\left(p\right)}{\bar{F}}_{X}\left(x\right)\text{d}x=E\left[\min\left\{X,F_{X}^{-1}\left(p\right)\right\}\right],\\ T_{Y}\left(p\right)\equiv \int_{0}^{G_{Y}^{-1}\left(p\right)}{\bar{G}}_{Y}\left(x\right)\text{d}x=E\left[\min\left\{Y,G_{Y}^{-1}\left(p\right)\right\}\right], \end{array}$$

that is, *T*_*X*_(*p*) and *T*_*Y*_(*p*) are the mean service times of *X* and *Y* before the respective equal probability time points. We wish to compare *T*_*X*_(*p*) and *T*_*Y*_(*p*) by means of a ratio *T*_*Y*_(*p*)/*T*_*X*_(*p*) Based on such an idea, we give Definition 4.

Let *X* and *Z* be two nonnegative continuous random variables with respective distribution functions *F*_*X*_ and *H*_*Z*_ such that *F*_*X*_(0)=*H*_*Z*_(0)=0, right-continuous inverse functions *F*_*X*_^−1^ and *H*_*Z*_^−1^, survival functions ${\bar{F}}_{X}$ and ${\bar{H}}_{Z}$, and density functions *f*_*X*_ and *h*_*Z*_, respectively.

**Definition 4 .**

*X* is said to be smaller than *Z* in the decreasing mean time to failure (DMTFR) order (denoted by *X*≤_dmtfr_*Z*), if

(19) $$\begin{array}{c} \frac{\int_{0}^{H_{Z}^{-1}\left(p\right)}{\bar{H}}_{Z}\left(x\right)\text{d}x}{\int_{0}^{F_{X}^{-1}\left(p\right)}{\bar{F}}_{X}\left(x\right)\text{d}x}, \end{array}$$

is increasing in *p* ∈ (0,1).

From Definition 3, the following lemma is obvious, the proof is omitted here.

**Lemma 2 .**

The following statements are equivalent:

(1) *X*≤_dmtfr_*Z*.
(2) The function *T*_*Z*_(*p*)/*T*_*X*_(*p*) is increasing in *p* ∈ (0,1).
(3) The inequality
  (20) $$\begin{array}{c} \int_{0}^{t}{\bar{F}}_{X}\left(x\right)\left[\frac{f_{X}\left(t\right)}{h_{Z}\left[H_{Z}^{-1}\left(F_{X}\left(t\right)\right)\right]}-\frac{f_{X}\left(x\right)}{h_{Z}\left[H_{Z}^{-1}\left(F_{X}\left(x\right)\right)\right]}\right]\text{d}x\geq 0,\;\text{for all }t>0, \end{array}$$
is valid.
(4) The inequality
  (21) $$\begin{array}{c} t{\bar{F}}_{X}\left(t\right)\left[\frac{f_{X}\left(t\right)}{h_{Z}\left[H_{Z}^{-1}\left(F_{X}\left(t\right)\right)\right]}-\frac{H_{Z}^{-1}\left(F_{X}\left(t\right)\right)}{t}\right]-\\ \int_{0}^{t}x\left(\frac{f_{X}\left(t\right)}{h_{Z}\left[H_{Z}^{-1}\left(F_{X}\left(t\right)\right)\right]}-\frac{H_{Z}^{-1}\left(F_{X}\left(x\right)\right)}{x}\right)d\left[{\bar{F}}_{X}\left(x\right)\right]\geq 0,\;\text{for all }t>0, \end{array}$$
is valid.

Shaked and Shanthikumar [8] studied the generalized TTT transform and proposed the generalized TTT transform order. Bartoszewicz and Benduch [29] further studied some properties of the generalized TTT transform by iteration. Motivated by their excellent works, we establish the following two new stochastic orders, the RDMTFR and the GDMTFR orders, see Definitions 5 and 6, respectively.

**Definition 5 .**

Let *X*, *Z*, and *Y* be three random variables. *X* is said to be smaller than *Z* relative to *Y* in the relative DMTFR order (denoted by *X*≤_rdmtfr_*Z* r.t. *Y*), if

(22) $$\begin{array}{c} \frac{\int_{0}^{H_{Z}^{-1}\left(p\right)}g_{Y}\left[G_{Y}^{-1}\left(H_{Z}\left(x\right)\right)\right]\text{d}x}{\int_{0}^{F_{X}^{-1}\left(p\right)}g_{Y}\left[G_{Y}^{-1}\left(F_{X}\left(x\right)\right)\right]\text{d}x}, \end{array}$$

is increasing in *p* ∈ (0,1).

From Definition 5, the following lemma is obvious, and the proof is omitted here.

**Lemma 3 .**

The following statements are equivalent:

(1) *X*≤_rdmtfr_*Z* r.t. *Y*.
(2) The function *T*_*Z*_(*p*; *Y*)/*T*_*X*_(*p*; *Y*) is increasing in *p* ∈ (0,1).
(3) The inequality
  (23) $$\begin{array}{c} \int_{0}^{t}g_{Y}\left[G_{Y}^{-1}\left(F_{X}\left(x\right)\right)\right]\left[\frac{f_{X}\left(t\right)}{h_{Z}\left[H_{Z}^{-1}\left(F_{X}\left(t\right)\right)\right]}-\frac{f_{X}\left(x\right)}{h_{Z}\left[H_{Z}^{-1}\left(F_{X}\left(x\right)\right)\right]}\right]\text{d}x\geq 0,\;\text{for all }t>0, \end{array}$$
is valid.
(4) The inequality
  (24) $$\begin{array}{c} tg_{Y}\left[G_{Y}^{-1}\left(F_{X}\left(t\right)\right)\right]\left[\frac{f_{X}\left(t\right)}{h_{Z}\left[H_{Z}^{-1}\left(F_{X}\left(t\right)\right)\right]}-\frac{H_{Z}^{-1}\left(F_{X}\left(t\right)\right)}{t}\right]-\\ \int_{0}^{t}x\left(\frac{f_{X}\left(t\right)}{h_{Z}\left[H_{Z}^{-1}\left(F_{X}\left(t\right)\right)\right]}-\frac{H_{Z}^{-1}\left(F_{X}\left(x\right)\right)}{x}\right)\text{d}\left[g_{Y}\left[G_{Y}^{-1}\left(F_{X}\left(x\right)\right)\right]\right]\geq 0,\;\text{for all }t>0, \end{array}$$
is valid.

It can be easily seen that when *Y* is an exponential random variable with a rate *λ* > 0, that is, *Y* ~ *𝔼*(*λ*), there exists a simple relationship between the relative DMTFR order and the DMTFR order:

(25) $$\begin{array}{c} X\leq_{\text{rdmtfr}}Z\;r.t.\;Y\;⟺\;X\leq_{\text{dmtfr}}Z. \end{array}$$

Assume that *ψ* is a real function defined on (0,1). Denote by

(26) $$\begin{array}{c} T_{X}\left(p;\psi \right)=\int_{0}^{F_{X}^{-1}\left(p\right)}\psi \left[F_{X}\left(x\right)\right]\text{d}x,\\ T_{Z}\left(p;\psi \right)=\int_{0}^{H_{Z}^{-1}\left(p\right)}\psi \left[H_{Z}\left(x\right)\right]\text{d}x,\;\text{for all }p\in \left(0,1\right). \end{array}$$

*T*_*X*_(*p*; *ψ*) and *T*_*Z*_(*p*; *ψ*) are called the generalized TTT transforms of *X* and *Z* relative to *ψ*, respectively. Then, we give the following definition.

**Definition 6 .**

Let *X* and *Z* be two absolutely continuous nonnegative random variables; assume that *ψ* is a real function defined on (0,1). *X* is said to be smaller than *Z* with respect to *ψ* in the generalized DMTFR order (denoted by *X*≤_gdmtfr_*Z* w.r.t. *ψ*), if the function

(27) $$\begin{array}{c} \frac{\int_{0}^{H_{Z}^{-1}\left(p\right)}\psi \left[H_{Z}\left(x\right)\right]\text{d}x}{\int_{0}^{F_{X}^{-1}\left(p\right)}\psi \left[F_{X}\left(x\right)\right]\text{d}x}, \end{array}$$

is increasing in *p* ∈ (0,1).

**Remark 1 .**

One can verify that the GDMTFR order is a partial order relation. The reflexive and transitive are evident; the antisymmetric is as follows (see Theorem 1 (30) as follows).

*X*≤_gdmtfr_*Z* w.r.t. *ψ* and *Z*≤_gdmtfr_*X* w.r.t. *ψ* hold simultaneously, if and only if *Y*=*kX*+*b* almost surely, where *k* and *b* are any real numbers such that *Y* is nonnegative and *k* ≠ 0.

**Remark 2 .**

It can be seen that the GDMTFR and DMRL orders do not have necessary implication relations each other. Especially, the DMTFR and DMRL orders do not have direct implication relationships each other.

From Definition 5, the following lemma is obvious, and the proof is omitted here.

It can be easily seen that when *ψ*(*u*)=*g*_*Y*_[*G*_*Y*_^−1^(*u*)], *u* ∈ (0,1), there exists a simple relationship between the GDMTFR order and the RDMTFR order:

(28) $$\begin{array}{c} X\leq_{\text{gdmtfr}}Z\text{w}.\text{r}.\text{t}.\psi \;⟺\;X\leq_{\text{rdmtfr}}Z\text{r}.\text{t}.Y. \end{array}$$

Now, we consider a system composed of same components. We say that the system preserves some properties if the components of this system possess some properties, and we conclude from the structure of the system that the system also possesses the same property. And conversely, we say that this system has the reversed preservation for some property if the system has some property. According to the structure of the system, we conclude that the components of this system also have the same property.

For two systems, if their two components satisfy some stochastic order relation, by the structure of these two systems, we conclude that the two systems also satisfy the same stochastic order relation; then, we say that these systems of the structure preserve this stochastic order relation, or equivalently, we say that this stochastic order relation possesses closure property under the structure. Conversely, if two systems satisfy some stochastic order relation, by the structure of these two systems, we conclude that the two components of the two systems also satisfy the same stochastic order relation, and then we say that these systems of the structure reversely preserve this stochastic order relation, or equivalently, we say that this stochastic order relation possesses reversed closure property under the structure.

In reliability theory, such two problems are often of interest: one is to investigate the closure properties of a stochastic order, and the other is to examine the reversed closure properties of a stochastic order under several reliability operations, such as increasing convex transforms and taking of maxima and minima.

To prove our main results, we first introduce the following lemma from Barlow and Proschan [18], which plays a key role in the proofs of this paper and are repeatedly used in the sequel.

**Lemma 4 .**

Let *W* be a measure on the interval (*a*, *b*), not necessarily nonnegative, where −*∞* ≤ *a* < *b* ≤ +*∞*. Let *h* be a nonnegative function defined on (*a*, *b*). If ∫_*a*_^*t*^d*W*(*x*) ≥ 0. for all *t* ∈ (*a*, *b*) and if *h* is decreasing, then

(29) $$\begin{array}{c} \int_{a}^{b}h\left(x\right)\text{d}W\left(x\right)\geq 0. \end{array}$$

In the next, we assume that all the random variables, under consideration, are nonnegative and continuous.

Throughout this paper, the term *increasing* stands for monotone nondecreasing and *decreasing* stands for monotone nonincreasing. Assume that all the random variables under considerations are nonnegative and absolutely continuous with 0 as the left endpoint of their supports, and that all the integrals and expectations involved are always finite. All the encountered ratios are always supposed to be well defined.

In this paper, we devote our interest to the closure properties of the GDMTFR order. The paper is organized as follows. First, in Section 2, we consider characterizations of GDMTFR order. We investigate the implication relationships between DMTFR and GDMTFR orders in Section 3. The closure and reversed closure properties of the GDMTFR order are studied in Section 4. Finally, in Section 5, we give two examples which meet the GDMTFR order.

In the following, we always assume that *X* and *Z* are two absolutely continuous and nonnegative random variables with distribution functions *F*_*X*_ and *H*_*Z*_ such that *F*_*X*_(0)=*H*_*Z*_(0)=0, survival functions ${\bar{F}}_{X}\equiv 1-F$ and ${\bar{H}}_{Z}\;\equiv 1-H_{Z}$, density functions *f*_*X*_ and *h*_*Z*_, and right-continuous inverse functions *F*_*X*_^−1^ and *H*_*Z*_^−1^ of *F*_*X*_ and *H*_*Z*_, respectively.

## 2. Characterizations of the GDMTFR Order

Now, we explore some characterizations of the GDMTFR order. First, we give a result by Definition 1.6, which will be useful in the proofs of upcoming theorems.

**Theorem 1 .**

The following statements are equivalent:

(1) *X*≤_gdmtfr_*Z* w.r.t. *ψ*.
(2) The inequality
  (30) $$\begin{array}{c} \int_{0}^{t}\psi \left[F_{X}\left(x\right)\right]\left[\frac{f_{X}\left(t\right)}{h_{Z}\left[H_{Z}^{-1}\left(F_{X}\left(t\right)\right)\right]}-\frac{f_{X}\left(x\right)}{h_{Z}\left[H_{Z}^{-1}\left(F_{X}\left(x\right)\right)\right]}\right]\text{d}x\geq 0,\;\text{for all }t>0, \end{array}$$
is valid.
(3) The inequality
  (31) $$\begin{array}{c} t\psi \left[F_{X}\left(t\right)\right]\left[\frac{f_{X}\left(t\right)}{h_{Z}\left[H_{Z}^{-1}\left(F_{X}\left(t\right)\right)\right]}-\frac{H_{Z}^{-1}\left(F_{X}\left(t\right)\right)}{t}\right]\\ -\int_{0}^{t}x\left(\frac{f_{X}\left(t\right)}{h_{Z}\left[H_{Z}^{-1}\left(F_{X}\left(t\right)\right)\right]}-\frac{H_{Z}^{-1}\left(F_{X}\left(x\right)\right)}{x}\right)\text{d}\left[\psi \left(F_{X}\left(x\right)\right)\right]\geq 0,\;\text{for all }t>0, \end{array}$$
is valid.

**Proof**

We only give proof for the case of (1)⟺(2). Suppose that the function

(32) $$\begin{array}{c} \frac{\int_{0}^{H_{Z}^{-1}\left(p\right)}\psi \left[H_{Z}\left(x\right)\right]\text{d}x}{\int_{0}^{F_{X}^{-1}\left(p\right)}\psi \left[F_{X}\left(x\right)\right]\text{d}x}, \end{array}$$

is increasing in *p* ∈ (0,1). By differentiating, we have that the numerator of the derivative of this ratio is

(33) $$\begin{array}{c} \frac{\psi \left(p\right)}{h_{Z}\left[H_{Z}^{-1}\left(p\right)\right]}\int_{0}^{F_{X}^{-1}\left(p\right)}\psi \left[F_{X}\left(x\right)\right]\text{d}x-\frac{\psi \left(p\right)}{f_{X}\left[F_{X}^{-1}\left(p\right)\right]}\int_{0}^{H_{Z}^{-1}\left(p\right)}\psi \left[H_{Z}\left(x\right)\right]\text{d}x,\;\text{for all }p\in \left(0,1\right). \end{array}$$

Since the function

(34) $$\begin{array}{c} \frac{\int_{0}^{H_{Z}^{-1}\left(p\right)}\psi \left[H_{Z}\left(x\right)\right]\text{d}x}{\int_{0}^{F_{X}^{-1}\left(p\right)}\psi \left[F_{X}\left(x\right)\right]\text{d}x}, \end{array}$$

is increasing in *p* ∈ (0,1), we have

(35) $$\begin{array}{c} \int_{0}^{F_{X}^{-1}\left(p\right)}\frac{f_{X}\left[F_{X}^{-1}\left(p\right)\right]}{h_{Z}\left[H_{Z}^{-1}\left(p\right)\right]}\psi \left[F_{X}\left(x\right)\right]\text{d}x-\int_{0}^{H_{Z}^{-1}\left(p\right)}\psi \left[H_{Z}\left(x\right)\right]\text{d}x\geq 0,\;\text{for all }p\in \left(0,1\right). \end{array}$$

Letting *H*_*Z*_(*x*)=*F*_*X*_(*y*) in the second integral of the left-hand side of inequality (35) and then letting *F*_*X*_^−1^(*p*)=*t*, we obtain that

(36) $$\begin{array}{c} \int_{0}^{t}\psi \left[F_{X}\left(x\right)\right]\left[\frac{f_{X}\left(t\right)}{h_{Z}\left[H_{Z}^{-1}\left(F_{X}\left(t\right)\right)\right]}-\frac{f_{X}\left(x\right)}{h_{Z}\left[H_{Z}^{-1}\left(F_{X}\left(x\right)\right)\right]}\right]\text{d}x\geq 0,\;\text{for all }t>0. \end{array}$$

And, the above deduction is reversible. Therefore, the proof of the theorem is complete.

**Definition 7 .**

Let *X* and *Z* be nonnegative absolutely continuous random variables with distribution functions *F*_*X*_, *H*_*Z*_ such that *F*_*X*_(0)=*H*_*Z*_(0)=0, density functions *f*_*X*_, *h*_*Z*_, and right-continuous inverse functions *F*_*X*_^−1^, *H*_*Z*_^−1^, respectively.

1. *X* is said to be smaller than *Z* in the starshaped order (denoted by *X*≤_*∗*_*Z*), if the function *H*_*Z*_^−1^(*F*_*X*_(*x*))/*x* is increasing in *x* > 0.
2. *X* is said to be smaller than *Z* in the convex order (denoted by *X*≤_c_*Z*), if the function *H*_*Z*_^−1^(*F*_*X*_(*x*)) is convex for *x* > 0.
3. *X* is said to be smaller than *Z* in the dispersive order (denoted by *X*≤_disp_*Z*), if *f*_*X*_[*F*_*X*_^−1^(*p*)] ≥ *h*_*Z*_[*H*_*Z*_^−1^(*p*)] for all *p* ∈ (0,1).

**Theorem 2 .**

Let *ψ* be a nonnegative function defined on (0,1). Then, for any *θ* > 0, *X*≤_gdmtfr_*θX* w.r.t. *ψ*.

**Theorem 3 .**

Let *ψ* be a nonnegative function defined on (0,1). If *X*≤_gdmtfr_*Z* w.r.t. *ψ*, then for any *θ* > 0, *X*≤_gdmtfr_*θZ* w.r.t. *ψ*.

**Proof**

Suppose that *X*≤_gdmtfr_*Z* w.r.t. *ψ*, then from inequality (30), we have

(37) $$\begin{array}{c} \int_{0}^{t}\psi \left[F_{X}\left(x\right)\right]\left[\frac{f_{X}\left(t\right)}{h_{Z}\left[H_{Z}^{-1}\left(F_{X}\left(t\right)\right)\right]}-\frac{f_{X}\left(x\right)}{h_{Z}\left[H_{Z}^{-1}\left(F_{X}\left(x\right)\right)\right]}\right]\text{d}x\geq 0,\;\text{for all }t>0. \end{array}$$

In turn, from (30), *X*≤_gdmtfr_*θZ* w.r.t. *ψ* if and only if, for all  *t* > 0,

(38) $$\begin{array}{c} \int_{0}^{t}\psi \left[F_{X}\left(x\right)\right]\left[\frac{f_{X}\left(t\right)}{h_{\theta Z}\left[H_{\theta Z}^{-1}\left(F_{X}\left(t\right)\right)\right]}-\frac{f_{X}\left(x\right)}{h_{\theta Z}\left[H_{\theta Z}^{-1}\left(F_{X}\left(x\right)\right)\right]}\right]\text{d}x\\ =\theta \int_{0}^{t}\psi \left[F_{X}\left(x\right)\right]\left[\frac{f_{X}\left(t\right)}{h_{Z}\left[H_{Z}^{-1}\left(F_{X}\left(t\right)\right)\right]}-\frac{f_{X}\left(x\right)}{h_{Z}\left[H_{Z}^{-1}\left(F_{X}\left(x\right)\right)\right]}\right]\text{d}x\geq 0. \end{array}$$

Clearly, if inequality (37) holds, then inequality (38) also holds, that is, *X*≤_gdmtfr_*θZ* w.r.t. *ψ*. Therefore, the proof of the theorem is complete.

**Remark 3 .**

Theorem 3 states that the order ≤_gdmtfr_ is scale invariant with respect to the compared variables.

**Theorem 4 .**

If *X*≤_gdmtfr_*Z* w.r.t. *ψ*, then for any *a* > 0, *X*≤_gdmtfr_*Z* w.r.t. *aψ*.

**Remark 4 .**

Theorem 4 states that the ≤_gdmtfr_ order is scale invariant with respect to the referred function.

**Theorem 5 .**

If *X*≤_gdmtfr_*Z* w.r.t. *ψ*, then for any *θ* > 0, *θX*≤_gdmtfr_*θZ* w.r.t. *ψ*.

**Remark 5 .**

Theorem 5 states that the order ≤_gdmtfr_ is scale equivalent with respect to the compared random variables.

**Theorem 6 .**

If *X*≤_gdmtfr_*Z* w.r.t. *ψ*, then for any *θ* > 0 and *a* > 0, *X*≤_gdmtfr_*θZ* w.r.t. *aψ*.

**Remark 6 .**

Theorem 6 states that the order ≤_gdmtfr_ is scale invariant with respect to the compared random variables and the referred random variable.

**Theorem 7 .**

Let *H*_*Z*_(0)=*F*_*X*_(0)=0. *X*≤_gdmtfr_*Z* w.r.t. *ψ* and *X*≥_gdmtfr_*Z* w.r.t. *ψ* if and only if there exists some *θ* > 0 such that *F*_*X*_(*x*)=*H*_*Z*_(*θx*), that is, $Z\overset{d}{=}\theta X$, where “$\overset{\text{d}}{=}$” means equality in distributions.

**Proof**

Suppose that *X*≤_gdmtfr_*Z* w.r.t. *ψ* and *X*≥_gdmtfr_*Z* w.r.t. *ψ*. From Definition 6, we have that there exists some *θ* > 0 such that

(39) $$\begin{array}{c} \frac{\int_{0}^{H_{Z}^{-1}\left(p\right)}\psi \left[H_{Z}\left(x\right)\right]\text{d}x}{\int_{0}^{F_{X}^{-1}\left(p\right)}\psi \left[F_{X}\left(x\right)\right]\text{d}x}=\theta . \end{array}$$

That is,

(40) $$\begin{array}{c} \int_{0}^{H_{Z}^{-1}\left(p\right)}\psi \left[H_{Z}\left(x\right)\right]\text{d}x=\theta \int_{0}^{F_{X}^{-1}\left(p\right)}\psi \left[F_{X}\left(x\right)\right]\text{d}x. \end{array}$$

Differentiating both sides of above equality, we obtain (*H*_*Z*_^−1^(*p*))′=(*F*_*θX*_^−1^(*p*))′. Thus, *H*_*Z*_^−1^(*p*)=*F*_*θX*_^−1^(*p*)+*c*, where *c* is any real number. By the assumption of *H*_*Z*_(0)=*F*_*X*_(0)=0, *c*=0 and then *H*_*Z*_^−1^(*p*)=*F*_*θX*_^−1^(*p*). Hence, $Z\overset{\text{d}}{=}\theta X$, and this is the stated result.

The following theorem gives a sufficient condition for the GDMTFR order.

**Theorem 8 .**

Let *ψ* be a nonnegative function defined on (0,1). If *X*≤_c_*Z*, then *X*≤_gdmtfr_*Z* w.r.t. *ψ*.

**Proof**

From (30) *X*≤_gdmtfr_*Z* w.r.t. *ψ* if and only if

(41) $$\begin{array}{c} \int_{0}^{t}\psi \left[F_{X}\left(x\right)\right]\left[\frac{f_{X}\left(t\right)}{h_{Z}\left[H_{Z}^{-1}\left(F_{X}\left(t\right)\right)\right]}-\frac{f_{X}\left(x\right)}{h_{Z}\left[H_{Z}^{-1}\left(F_{X}\left(x\right)\right)\right]}\right]\text{d}x\geq 0,\;\text{for all }t>0. \end{array}$$

If *X*≤_c_*Z*, then the function *f*_*X*_(*x*)/*g*_*Y*_[*G*_*Y*_^−1^(*F*_*X*_(*x*))] is increasing; hence,

(42) $$\begin{array}{c} \frac{f_{X}\left(x\right)}{h_{Z}\left[H_{Z}^{-1}\left(F_{X}\left(x\right)\right)\right]}\leq \frac{f_{X}\left(t\right)}{h_{Z}\left[H_{Z}^{-1}\left(F_{X}\left(t\right)\right)\right]},\;\text{for all }0\leq x\leq t. \end{array}$$

Moreover, *ψ*[*F*_*X*_(*x*)] ≥ 0 for all  *x* ≥ 0. From inequality (42), we see that inequality (41) holds. That is, *X*≤_gdmtfr_*Z* w.r.t. *ψ*. Therefore, the proof of the theorem is complete.

## 3. Implication Relationships between DMTFR and GDMTFR Orders

Recall from [5] that a function *h* : (0,1)⟶(0,1) is called star-shaped (anti-star-shaped) if *h*(*x*)/*x* is increasing (decreasing). *h* is star-shaped (anti-star-shaped) with respect to the point (1,1) if [1 − *h*(*x*)]/[1 − *x*] is increasing (decreasing) in *x*.

Now, we propose the following notions about real functions.

**Definition 8 .**

For a positive integer number *n*, we say that

1. *h* is star-shaped (anti-star-shaped) with respect to the point (1,0) if *h*(*x*)/(1 − *x*) is decreasing (increasing).
2. *h* is dual (anti-star-shaped) star-shaped of order *n* if *h*(*x*)/(1 − *x*^*n*^) is increasing (decreasing).
3. *h* is star-shaped (anti-star-shaped) of order *n* with respect to the point (1,0) if *h*(*x*)/(1 − *x*)^*n*^ is decreasing (increasing) in *x* ∈ (0,1).

Let *X* and *Z* be two nonnegative continuous random variables with respective distribution functions *F*_*X*_ and *H*_*Z*_ having 0 as the common left endpoint of their supports. Let again *ψ* be a nonnegative function defined on (0,1). Assume that *X*_1_,…, *X*_*n*_ and *Z*_1_,…, *Z*_*n*_ are the i.i.d. copies of *X* and *Z*, respectively, and that *X*_*k*:*n*_ and *Z*_*k*:*n*_, *k*=1,2,…, *n*, are the order statistics of *X* and *Z*, respectively. Let *X*_*k*:*n*_ have distribution function *F*_*X*_*k*:*n*__(*x*), survival function ${\bar{F}}_{X_{k:n}}\left(x\right)$, and density function *f*_*X*_*k*:*n*__(*x*), then for all *x* ≥ 0,

(43) $$\begin{array}{c} F_{X_{k:n}}\left(x\right)=B_{k,n-k+1}\left(F_{X}\left(x\right)\right),\\ {\bar{F}}_{X_{k:n}}\left(x\right)=B_{n-k+1,k}\left({\bar{F}}_{X}\left(x\right)\right),\\ f_{X_{k:n}}\left(x\right)=\beta_{k,n-k+1}\left(F_{X}\left(x\right)\right)f_{X}\left(x\right)=\frac{n!}{\left(k-1\right)!\left(n-k\right)!}{\left[F_{X}\left(x\right)\right]}^{k-1}{\left[{\bar{F}}_{X}\left(x\right)\right]}^{n-k}f_{X}\left(x\right), \end{array}$$

where *B*_*k*,*n*−*k*+1_(·) and *β*_*k*,*n*−*k*+1_(·) are the distribution function and density function of a beta distribution with parameters *k* and *n* − *k*+1, respectively.

The following Theorem 9 gives some conditions under which the DMTFR order implies the GDMTFR order.

**Theorem 9 .**

(1) Assume that the function *ψ* ° *B*_*k*,*n*−*k*+1_ is star-shaped with respect to the point (1,0). If *X*≤_dmtfr_*Z*, then
  (44) $$\begin{array}{c} X_{k:n}\leq_{\text{gdmtfr}}Z_{k:n}w.r.t.\psi ,\;\text{for all }k=1,2,\ldots ,n, \end{array}$$
where *ψ*°*B*_*k*,*n*−*k*+1_ is the compound of *ψ* and *B*_*k*,*n*−*k*+1_.
(2) Assume that the function *ψ* is star-shaped with respect to the point (1,0). If *X*≤_dmtfr_*Z*, then
  (45) $$\begin{array}{c} X\leq_{\text{gdmtfr}}Z\;w.r.t.\psi . \end{array}$$
(3) Assume that the function *ψ* is star-shaped of order *n* with respect to the point (1,0). If *X*_1:*n*_≤_dmtfr_*Z*_1:*n*_, then
  (46) $$\begin{array}{c} X\leq_{\text{gdmtfr}}Zw.r.t.\psi . \end{array}$$
(4) Assume that the function *ψ* is dual anti-star-shaped of order *n*. If *X*_*n*:*n*_≤_dmtfr_*Z*_*n*:*n*_, then
  (47) $$\begin{array}{c} X\leq_{\text{gdmtfr}}Z\text{ w. r. t. }\psi . \end{array}$$
(5) Assume that the function *ψ* star-shaped with respect to the point (1,0). If for some 1 ≤ *k* ≤ *n*, *X*_*k*:*n*_≤_dmtfr_*Z*_*k*:*n*_, then
  (48) $$\begin{array}{c} X_{k:n}\leq_{\text{gdmtfr}}Z_{k:n}w.r.t.\psi ,\;\text{for all }k=1,2,\ldots ,n. \end{array}$$

**Proof**

Barlow and Proschan [18] proved that, for all *x* ≥ 0,

(49) $$\begin{array}{c} H_{Z_{k:n}}^{-1}\left[F_{X_{k:n}}\left(x\right)\right]={\bar{H}}_{Z_{k:n}}^{-1}\left[{\bar{F}}_{X_{k:n}}\left(x\right)\right]=H_{Z}^{-1}\left[F_{X}\left(x\right)\right]. \end{array}$$

It can be proven that

(50) $$\begin{array}{c} \frac{f_{X_{k:n}}\left(x\right)}{h_{Z_{k:n}}\left[H_{Z_{k:n}}^{-1}\left(F_{X_{k:n}}\left(x\right)\right)\right]}\frac{f_{X}\left(x\right)}{h_{Z}\left[H_{Z}^{-1}\left(F_{X}\left(x\right)\right)\right]}. \end{array}$$

(1) Suppose that *X*≤_dmtfr_*Z*. Then, we have, for all *t* > 0,
  (51) $$\begin{array}{c} \int_{0}^{t}{\bar{F}}_{X}\left(x\right)\left[\frac{f_{X}\left(t\right)}{h_{Z}\left[H_{Z}^{-1}\left(F_{X}\left(t\right)\right)\right]}-\frac{f_{X}\left(x\right)}{h_{Z}\left[H_{Z}^{-1}\left(F_{X}\left(x\right)\right)\right]}\right]\text{d}x\\ =\int_{0}^{t}{\bar{F}}_{X}\left(x\right)\left[\frac{f_{X_{k:n}}\left(t\right)}{h_{Z_{k:n}}\left[H_{Z_{k:n}}^{-1}\left(F_{X_{k:n}}\left(t\right)\right)\right]}-\frac{f_{X_{k:n}}\left(x\right)}{h_{Z_{k:n}}\left[H_{Z_{k:n}}^{-1}\left(F_{X_{k:n}}\left(x\right)\right)\right]}\right]\text{d}x\geq 0. \end{array}$$
Since *ψ* ° *B*_*k*,*n*−*k*+1_ is star-shaped with respect to the point (1,0), then *ψ*°*B*_*k*,*n*−*k*+1_(*u*)/(1 − *u*) is decreasing in *u* ∈ (0,1). Hence, the function
  (52) $$\begin{array}{c} h\left(x\right)=\psi {}^{\circ}B_{k,n-k+1}\frac{\left[F_{X}\left(x\right)\right]}{\left(1-F_{X}\left(x\right)\right)}=\psi \frac{\left[F_{X_{k:n}}\left(x\right)\right]}{{\bar{F}}_{X}\left(x\right)}, \end{array}$$
is nonnegative decreasing in *x* ≥ 0. From Lemma 4 and inequality (51), we have
  (53) $$\begin{array}{c} \int_{0}^{t}\psi \left[F_{X_{k:n}}\left(x\right)\right]\left[\;\frac{f_{X_{k:n}}\left(t\right)}{h_{Z_{k:n}}\left[H_{Z_{k:n}}^{-1}\left(F_{X_{k:n}}\left(t\right)\right)\right]}-\frac{f_{X_{k:n}}\left(x\right)}{h_{Z_{k:n}}\left[H_{Z_{k:n}}^{-1}\left(F_{X_{k:n}}\left(x\right)\right)\right]}\right]\text{d}x\geq 0,\;\text{for all }t>0, \end{array}$$
which is equivalent to
  (54) $$\begin{array}{c} X_{k:n}\leq_{\text{gdmtfr}}Z_{k:n}\;w.r.t.\psi ,\;\text{for all }k=1,2,\ldots ,n. \end{array}$$
(2) Suppose that *X*≤_dmtfr_*Z*. Then, for all *t* > 0,
  (55) $$\begin{array}{c} \int_{0}^{t}{\bar{F}}_{X}\left(x\right)\left[\frac{f_{X}\left(t\right)}{h_{Z}\left[H_{Z}^{-1}\left(F_{X}\left(t\right)\right)\right]}-\frac{f_{X}\left(x\right)}{h_{Z}\left[H_{Z}^{-1}\left(F_{X}\left(x\right)\right)\right]}\right]\text{d}x\geq 0. \end{array}$$
Since the function *ψ* is star-shaped with respect to the point (1,0), the function *ψ*(*u*)/(1 − *u*) is decreasing in *u* ∈ (0,1). Thus, $h\left(x\right)=\psi \left[F_{X}\left(x\right)\right]/{\bar{F}}_{X}\left(x\right)$ is nonnegatively decreasing. From Lemma 4 and (55), we have
  (56) $$\begin{array}{c} \int_{0}^{t}\psi \left[F_{X}\left(x\right)\right]\left[\frac{f_{X}\left(t\right)}{h_{Z}\left[H_{Z}^{-1}\left(F_{X}\left(t\right)\right)\right]}-\frac{f_{X}\left(x\right)}{h_{Z}\left[H_{Z}^{-1}\left(F_{X}\left(x\right)\right)\right]}\right]\text{d}x\geq 0,\;\text{for all }t>0, \end{array}$$
which states that *X*≤_gdmtfr_*Z* w.r.t. *ψ*.
(3) Suppose that *X*_1:*n*_≤_dmtfr_*Z*_1:*n*_. Then, for all *t* > 0,
  (57) $$\begin{array}{c} \int_{0}^{t}{\bar{F}}_{X_{1:n}}\left(x\right)\left[\frac{f_{X_{1:n}}\left(t\right)}{h_{Z_{1:n}}\left[H_{Z_{1:n}}^{-1}\left(F_{X_{1:n}}\left(t\right)\right)\right]}-\;\frac{f_{X_{1:n}}\left(x\right)}{h_{Z_{1:n}}\left[H_{Z_{1:n}}^{-1}\left(F_{X_{1:n}}\left(x\right)\right)\right]}\right]\text{d}x\geq 0. \end{array}$$
Since *ψ* is star-shaped of order *n* with respect to the point (1,0), the function
  (58) $$\begin{array}{c} \frac{\psi \left(x\right)}{{\left(1-x\right)}^{n}}, \end{array}$$
is decreasing in *x*, which leads to the function
  (59) $$\begin{array}{c} h\left(x\right)=\frac{\psi \left[F_{X}\left(x\right)\right]}{{\left[1-F_{X}\left(x\right)\right]}^{n}}=\frac{\psi \left[F_{X}\left(x\right)\right]}{{\left[{\bar{F}}_{X}\left(x\right)\right]}^{n}}=\frac{\psi \left[F_{X}\left(x\right)\right]}{{\bar{F}}_{X_{1:n}}\left(x\right)}. \end{array}$$
being nonnegative decreasing. Moreover,
  (60) $$\begin{array}{c} \frac{f_{X_{1:n}}\left(x\right)}{h_{Z_{1:n}}\left[H_{Z_{1:n}}^{-1}\left(F_{X_{1:n}}\left(x\right)\right)\right]}=\frac{f_{X}\left(x\right)}{h_{Z}\left[H_{Z}^{-1}\left(F_{X}\left(x\right)\right)\right]}. \end{array}$$
From Lemma 4, (57), and (60), we have
  (61) $$\begin{array}{c} \int_{0}^{t}\psi \left[F_{X}\left(x\right)\right]\left[\frac{f_{X}\left(t\right)}{h_{Z}\left[H_{Z}^{-1}\left(F_{X}\left(t\right)\right)\right]}-\frac{f_{X}\left(x\right)}{h_{Z}\left[H_{Z}^{-1}\left(F_{X}\left(x\right)\right)\right]}\right]\text{d}x\geq 0,\;\text{for all }t>0, \end{array}$$
which is equivalent to
  (62) $$\begin{array}{c} X\leq_{\text{gdmtfr}}Z\;w.r.t.\psi . \end{array}$$
(4) Suppose that *X*_*n*:*n*_≤_dmtfr_*Z*_*n*:*n*_. Then, for all *t* > 0,
  (63) $$\begin{array}{c} \int_{0}^{t}F_{X_{n:n}}\left(x\right)\left[\;\frac{f_{X_{n:n}}\left(t\right)}{h_{Z_{n:n}}\left[H_{Z_{n:n}}^{-1}\left(F_{X_{n:n}}\left(t\right)\right)\right]}-\frac{f_{X_{n:n}}\left(x\right)}{h_{Z_{n:n}}\left[H_{Z_{n:n}}^{-1}\left(F_{X_{n:n}}\left(x\right)\right)\right]}\right]\text{d}x\geq 0. \end{array}$$
Since *ψ* is dual anti-star-shaped of order *n*, the function *ψ*(*x*)/(1 − *x*^*n*^) is nonnegative decreasing in *x* ∈ (0,1), which leads to the function
  (64) $$\begin{array}{c} h\left(x\right)=\frac{\psi \left[F_{X}\left(x\right)\right]}{1-{\left[F_{X}\left(x\right)\right]}^{n}}=\frac{\psi \left[F_{X}\left(x\right)\right]}{{\bar{F}}_{X_{n:n}}\left(x\right)}. \end{array}$$
being nonnegative decreasing. Moreover,
  (65) $$\begin{array}{c} \frac{f_{X_{n:n}}\left(x\right)}{h_{Z_{n:n}}\left[H_{Z_{n:n}}^{-1}\left(F_{X_{1:n}}\left(x\right)\right)\right]}=\frac{f_{X}\left(x\right)}{h_{Z}\left[H_{Z}^{-1}\left(F_{X}\left(x\right)\right)\right]}. \end{array}$$
From Lemma 4, (63)–(65), we have
  (66) $$\begin{array}{c} \int_{0}^{t}\psi \left[F_{X}\left(x\right)\right]\left[\frac{f_{X}\left(t\right)}{h_{Z}\left[H_{Z}^{-1}\left(F_{X}\left(t\right)\right)\right]}-\frac{f_{X}\left(x\right)}{h_{Z}\left[H_{Z}^{-1}\left(F_{X}\left(x\right)\right)\right]}\right]\text{d}x\geq 0,\;\text{for all }t>0, \end{array}$$
and this asserts that
  (67) $$\begin{array}{c} X\leq_{\text{gdmtfr}}Z\;w.r.t.\psi . \end{array}$$
(5) Let the function *ψ* be anti-star-shaped with respect to the point (1,0). Suppose that, for some 1 ≤ *k* ≤ *n*, *X*_*k*:*n*_≤_dmtfr_*Z*_*k*:*n*_, then for all *t* > 0,
  (68) $$\begin{array}{c} \int_{0}^{t}F_{X_{k:n}}\left(x\right)\left[\frac{f_{X_{k:n}}\left(t\right)}{h_{Z_{k:n}}\left[H_{Z_{k:n}}^{-1}\left(F_{X_{k:n}}\left(t\right)\right)\right]}-\frac{f_{X_{k:n}}\left(x\right)}{h_{Z_{k:n}}\left[H_{Z_{k:n}}^{-1}\left(F_{X_{k:n}}\left(x\right)\right)\right]}\right]\text{d}x\geq 0. \end{array}$$

Since the function *ψ* is star-shaped with respect to the point (1,0), the function *ψ*(*x*)/(1 − *x*) is nonnegatively decreasing in *x* ∈ (0,1). Thus, $\varphi \left(x\right)=\psi \left[F_{X_{k:n}}\left(x\right)\right]/{\bar{F}}_{X_{k:n}}\left(x\right)$ is nonnegatively decreasing. From Lemma 4 and (68), we have

(69) $$\begin{array}{c} \int_{0}^{t}\psi \left[F_{X_{k:n}}\left(x\right)\right]\left[\frac{f_{X_{k:n}}\left(t\right)}{h_{Z_{k:n}}\left[H_{Z_{k:n}}^{-1}\left(F_{X_{k:n}}\left(t\right)\right)\right]}-\frac{f_{X_{k:n}}\left(x\right)}{h_{Z_{k:n}}\left[H_{Z_{k:n}}^{-1}\left(F_{X_{k:n}}\left(x\right)\right)\right]}\right]\text{d}x\geq 0,\;\text{for all }t>0, \end{array}$$

which asserts that

(70) $$\begin{array}{c} X_{k:n}\leq_{\text{gdmtfr}}Z_{k:n}\;w.r.t.\psi ,\;\text{for all }k=1,2,\ldots ,n. \end{array}$$

Therefore, the proof of the theorem is complete.

Theorem 10 gives some conditions under which the GDMTFR order implies the DMTFR order.

**Theorem 10 .**

(1) Assume that the function *ψ* is anti-star-shaped with respect to the point (1,0). If for some 1 ≤ *k* ≤ *n*, *X*_*k*:*n*_≤_gdmtfr_*Z*_*k*:*n*_ w.r.t. *ψ*, then
  (71) $$\begin{array}{c} X_{k:n}\leq_{\text{dmtfr}}Z_{k:n},\;\text{for all }k=1,2,\ldots ,n. \end{array}$$
(2) Assume that the function *ψ* is anti-star-shaped with respect to the point (1,0). If *X*≤_gdmtfr_*Z* w.r.t. *ψ*, then
  (72) $$\begin{array}{c} X\leq_{\text{dmtfr}}Z. \end{array}$$
(3) Assume that the function *ψ* is dual star-shaped of order *n*. If *X*≤_gdmtfr_*Z* w.r.t. *ψ*, then
  (73) $$\begin{array}{c} X_{n:n}\leq_{\text{dmtfr}}Z_{n:n}. \end{array}$$
(4) Assume that the function *ψ* is anti-star-shaped of order *n* with respect to the point (1,0). If *X*≤_gdmtfr_*Z* w.r.t. *ψ*, then
  (74) $$\begin{array}{c} X_{1:n}\leq_{\text{dmtfr}}Z_{1:n}. \end{array}$$
(5) Assume that the function *ψ* is increasing in *u* ∈ (0,1). If *X*≤_dmtfr_*Z* w.r.t. *ψ*, then
  (75) $$\begin{array}{c} X_{k:n}\leq_{\text{gdmtfr}}Z_{k:n},\;\text{for all }k=1,2,\ldots ,n. \end{array}$$

**Proof**

(1) Suppose that, for some 1 ≤ *k* ≤ *n*, *X*_*k*:*n*_≤_gdmtfr_*Z*_*k*:*n*_ w.r.t. *ψ*. Then, for all *t* > 0,
  (76) $$\begin{array}{c} \int_{0}^{t}\psi \left[F_{X_{k:n}}\left(x\right)\right]\left[\frac{f_{X_{k:n}}\left(t\right)}{h_{Z_{k:n}}\left[H_{Z_{k:n}}^{-1}\left(F_{X_{k:n}}\left(t\right)\right)\right]}-\frac{f_{X_{k:n}}\left(x\right)}{h_{Z_{k:n}}\left[H_{Z_{k:n}}^{-1}\left(F_{X_{k:n}}\left(x\right)\right)\right]}\right]\text{d}x\geq 0. \end{array}$$
Since the function *ψ* is anti-star-shaped with respect to the point (1,0), the function (1 − *u*)/*ψ*(*u*) is nonnegatively decreasing in *u* ∈ (0,1). Thus, $h\left(x\right)={\bar{F}}_{X_{k:n}}\left(x\right)/\psi \left[F_{X_{k:n}}\left(x\right)\right]$ is nonnegatively decreasing. From Lemma 4 and (76), we have
  (77) $$\begin{array}{c} \int_{0}^{t}{\bar{F}}_{X_{k:n}}\left(x\right)\left[\frac{f_{X_{k:n}}\left(t\right)}{h_{Z_{k:n}}\left[H_{Z_{k:n}}^{-1}\left(F_{X_{k:n}}\left(t\right)\right)\right]}-\frac{f_{X_{k:n}}\left(x\right)}{h_{Z_{k:n}}\left[H_{Z_{k:n}}^{-1}\left(F_{X_{k:n}}\left(x\right)\right)\right]}\right]\text{d}x\geq 0,\;\text{for all }t>0, \end{array}$$
which asserts that
  (78) $$\begin{array}{c} X_{k:n}\leq_{\text{dmtfr}}Z_{k:n},\;\text{for all }k=1,2,\ldots ,n. \end{array}$$
(2) Suppose that *X*≤_gdmtfr_*Z* w.r.t. *ψ*. Then, for all *t* > 0,
  (79) $$\begin{array}{c} \int_{0}^{t}\psi \left[F_{X}\left(x\right)\right]\left[\frac{f_{X}\left(t\right)}{h_{Z}\left[H_{Z}^{-1}\left(F_{X}\left(t\right)\right)\right]}-\frac{f_{X}\left(x\right)}{h_{Z}\left[H_{Z}^{-1}\left(F_{X}\left(x\right)\right)\right]}\right]\text{d}x\geq 0. \end{array}$$
Since the function *ψ* is anti-star-shaped with respect to the point (1,0), the function (1 − *u*)/*ψ*(*u*) is decreasing in *u* ∈ (0,1). Thus, $h\left(x\right)={\bar{F}}_{X}\left(x\right)/\psi \left[F_{X}\left(x\right)\right]$ is nonnegatively decreasing. From Lemma 4 and (79), we have
  (80) $$\begin{array}{c} \int_{0}^{t}{\bar{F}}_{X}\left(x\right)\left[\frac{f_{X}\left(t\right)}{h_{Z}\left[H_{Z}^{-1}\left(F_{X}\left(t\right)\right)\right]}-\frac{f_{X}\left(x\right)}{h_{Z}\left[H_{Z}^{-1}\left(F_{X}\left(x\right)\right)\right]}\right]\text{d}x\geq 0,\;\text{for all }t>0, \end{array}$$
which states that
  (81) $$\begin{array}{c} X\leq_{\text{dmtfr}}Z. \end{array}$$
(3) Suppose that *X*≤_gdmtfr_*Z* w.r.t. *ψ*. Then, for all *t* > 0,
  (82) $$\begin{array}{c} \int_{0}^{t}\psi \left[F_{X}\left(x\right)\right]\left[\frac{f_{X}\left(t\right)}{h_{Z}\left[H_{Z}^{-1}\left(F_{X}\left(t\right)\right)\right]}-\frac{f_{X}\left(x\right)}{h_{Z}\left[H_{Z}^{-1}\left(F_{X}\left(x\right)\right)\right]}\right]\text{d}x\geq 0,\;\text{for all }t>0. \end{array}$$
Since *ψ* is dual star-shaped of order *n*, the function (1 − *u*^*n*^)/*ψ*(*u*) is nonnegative decreasing in *u* ∈ (0,1), which leads to the function
  (83) $$\begin{array}{c} \varphi \left(x\right)=\frac{1-{\left[F_{X}\left(x\right)\right]}^{n}}{\psi \left[F_{X}\left(x\right)\right]}=\frac{{\bar{F}}_{X_{n:n}}\left(x\right)}{\psi \left[F_{X}\left(x\right)\right]}, \end{array}$$
being nonnegative decreasing. Moreover,
  (84) $$\begin{array}{c} \frac{f_{X}\left(x\right)}{h_{Z}\left[H_{Z}^{-1}\left(F_{X}\left(x\right)\right)\right]}=\frac{f_{X_{n:n}}\left(x\right)}{h_{Z_{n:n}}\left[H_{Z_{n:n}}^{-1}\left(F_{X_{1:n}}\left(x\right)\right)\right]}. \end{array}$$
From Lemma 4, (82)–(84), we have
  (85) $$\begin{array}{c} \int_{0}^{t}{\bar{F}}_{X_{n:n}}\left(x\right)\left[\;\frac{f_{X_{n:n}}\left(t\right)}{h_{Z_{n:n}}\left[H_{Z_{n:n}}^{-1}\left(F_{X_{n:n}}\left(t\right)\right)\right]}-\;\frac{f_{X_{n:n}}\left(x\right)}{h_{Z_{n:n}}\left[H_{Z_{n:n}}^{-1}\left(F_{X_{n:n}}\left(x\right)\right)\right]}\right]\text{d}x\geq 0,\;\text{for all }t>0, \end{array}$$
and this asserts that
  (86) $$\begin{array}{c} X_{n:n}\leq_{\text{dmtfr}}Z_{n:n}. \end{array}$$
(4) Suppose that *X*≤_gdmtfr_*Z* w.r.t. *ψ*. Then, for all *t* > 0,
  (87) $$\begin{array}{c} \int_{0}^{t}\psi \left[F_{X}\left(x\right)\right]\left[\frac{f_{X}\left(t\right)}{h_{Z}\left[H_{Z}^{-1}\left(F_{X}\left(t\right)\right)\right]}-\frac{f_{X}\left(x\right)}{h_{Z}\left[H_{Z}^{-1}\left(F_{X}\left(x\right)\right)\right]}\right]\text{d}x\geq 0,\;\text{for all }t>0. \end{array}$$
Since *ψ* is anti-star-shaped of order *n* with respect to the point (1,0), the function (1 − *u*)^*n*^/*ψ*(*u*) is decreasing in *u* ∈ (0,1), and this leads to the function
  (88) $$\begin{array}{c} h\left(x\right)=\frac{{\left[1-F_{X}\left(x\right)\right]}^{n}}{\psi \left[F_{X}\left(x\right)\right]}=\frac{{\bar{F}}_{X_{1:n}}\left(x\right)}{\psi \left[F_{X}\left(x\right)\right]}, \end{array}$$
being nonnegatively decreasing. Moreover,
  (89) $$\begin{array}{c} \frac{f_{X_{1:n}}\left(x\right)}{h_{Z_{1:n}}\left[H_{Z_{1:n}}^{-1}\left(F_{X_{1:n}}\left(x\right)\right)\right]}=\frac{f_{X}\left(x\right)}{h_{Z}\left[H_{Z}^{-1}\left(F_{X}\left(x\right)\right)\right]}. \end{array}$$
From Lemma 4, (87)–(89), we have
  (90) $$\begin{array}{c} \int_{0}^{t}{\bar{F}}_{X_{1:n}}\left(x\right)\left[\;\frac{f_{X_{1:n}}\left(t\right)}{h_{Z_{1:n}}\left[H_{Z_{1:n}}^{-1}\left(F_{X_{1:n}}\left(t\right)\right)\right]}-\;\frac{f_{X_{1:n}}\left(x\right)}{h_{Z_{1:n}}\left[H_{Z_{1:n}}^{-1}\left(F_{X_{1:n}}\left(x\right)\right)\right]}\right]\text{d}x\geq 0,\;\text{for all }t>0, \end{array}$$
which is equivalent to
  (91) $$\begin{array}{c} X_{1:n}\leq_{\text{dmtfr}}Z_{1:n}. \end{array}$$
(5) Suppose that *X*≤_dmtfr_*Z* w.r.t. *ψ*. Then, we have, for all *t* > 0,
  (92) $$\begin{array}{c} \int_{0}^{t}\psi \left[F_{X}\left(x\right)\right]\left[\frac{f_{X}\left(t\right)}{h_{Z}\left[H_{Z}^{-1}\left(F_{X}\left(t\right)\right)\right]}-\frac{f_{X}\left(x\right)}{h_{Z}\left[H_{Z}^{-1}\left(F_{X}\left(x\right)\right)\right]}\right]\text{d}x\\ =\int_{0}^{t}\psi \left[F_{X}\left(x\right)\right]\left[\frac{f_{X_{k:n}}\left(t\right)}{h_{Z_{k:n}}\left[H_{Z_{k:n}}^{-1}\left(F_{X_{k:n}}\left(t\right)\right)\right]}-\frac{f_{X_{k:n}}\left(x\right)}{h_{Z_{k:n}}\left[H_{Z_{k:n}}^{-1}\left(F_{X_{k:n}}\left(x\right)\right)\right]}\right]\text{d}x\geq 0. \end{array}$$

Since *ψ* is increasing in *u* ∈ (0,1), then ${\bar{F}}_{X_{k:n}}\left(x\right)/\psi \left[F_{X}\left(x\right)\right]$ is nonnegatively decreasing in *x* > 0. From Lemma 4 and inequality (92), we have

(93) $$\begin{array}{c} \int_{0}^{t}\psi \left[F_{X_{k:n}}\left(x\right)\right]\left[\frac{f_{X_{k:n}}\left(t\right)}{h_{Z_{k:n}}\left[H_{Z_{k:n}}^{-1}\left(F_{X_{k:n}}\left(t\right)\right)\right]}-\frac{f_{X_{k:n}}\left(x\right)}{h_{Z_{k:n}}\left[H_{Z_{k:n}}^{-1}\left(F_{X_{k:n}}\left(x\right)\right)\right]}\right]\text{d}x\geq 0,\;\text{for all }t>0, \end{array}$$

which is equivalent to

(94) $$\begin{array}{c} X_{k:n}\leq_{\text{gdmtfr}}Z_{k:n},\;\text{for all }k=1,2,\ldots ,n. \end{array}$$

Therefore, the proof of the theorem is complete.

Denote by *X*_1:*N*_=min(*X*_1_, *X*_2_,…, *X*_*N*_) and *X*_*N*:*N*_=max(*X*_1_, *X*_2_,…, *X*_*N*_). *Z*_1:*N*_ and *Z*_*N*:*N*_ are similar. Let *φ*_*N*_(·) be the probability generating function of *N*.

**Theorem 11 .**

Assume that *X*_1_, *X*_2_,…, and *Z*_1_, *Z*_2_,…, are the i.i.d. copies of *X* and *Z*, respectively.

(1) Let the function *ψ*∘*φ*_*N*_ be star-shaped with respect to the point (1,0). If *X*≤_dmtfr_*Z*, then
  (95) $$\begin{array}{c} X_{N:N}\leq_{\text{gdmtfr}}Z_{N:N}\;w.r.t.\psi . \end{array}$$
(2) Let the function *ψ*∘*φ*_*N*_ be anti-star-shaped with respect to the point (1,0). If *X*_*N*:*N*_≤_gdmtfr_*Z*_*N*:*N*_ w.r.t. *ψ*, then
  (96) $$\begin{array}{c} X\leq_{\text{dmtfr}}Z. \end{array}$$
(3) Let *ψ* be a function defined on the interval (0,1) such that the function *ψ*(1 − *φ*_*N*_(*u*))/*u* is increasing in *u* ∈ (0,1). If *X*≤_dmtfr_*Z*, then
  (97) $$\begin{array}{c} X_{1:N}\leq_{\text{gdmtfr}}Z_{1:N}w.r.t.\psi . \end{array}$$
(4) Let *ψ* be a function defined on the interval (0,1) such that the function *u*/*ψ*(1 − *φ*_*N*_(*u*)) is increasing in *u* ∈ (0,1). If *X*_1:*N*_≤_gdmtfr_*Z*_1:*N*_ w.r.t. *ψ*, then
  (98) $$\begin{array}{c} X\leq_{\text{dmtfr}}Z. \end{array}$$

**Proof**

(1) Suppose that *X*≤_dmtfr_*Y*. This asserts that
  (99) $$\begin{array}{c} \int_{0}^{t}{\bar{F}}_{X}\left(x\right)\left[\frac{f_{X}\left(t\right)}{h_{Z}\left[H_{Z}^{-1}\left(F_{X}\left(t\right)\right)\right]}-\frac{f_{X}\left(x\right)}{h_{Z}\left[H_{Z}^{-1}\left(F_{X}\left(x\right)\right)\right]}\right]\text{d}x\geq 0,\;\text{for all }t>0. \end{array}$$
Shaked and Shanthikumar [8] showed that
  (100) $$\begin{array}{c} H_{Z_{N:N}}^{-1}\left(F_{X_{N:N}}\left(x\right)\right)=H_{Z}^{-1}\left(F_{X}\left(x\right)\right). \end{array}$$
It can be proven that
  (101) $$\begin{array}{c} \frac{f_{X_{N:N}}\left(x\right)}{h_{Z_{N:N}}\left[H_{Z_{N:N}}^{-1}\left(F_{X_{N:N}}\left(x\right)\right)\right]}=\frac{f_{X}\left(x\right)}{h_{Z}\left[H_{Z}^{-1}\left(F_{X}\left(x\right)\right)\right]}. \end{array}$$
Since the function *ψ*∘*φ*_*N*_ is star-shaped with respect to the point (1,0), the function *ψ*[*φ*_*N*_(*u*)]/(1 − *u*) is decreasing in *u* ∈ (0,1), and this leads to the function
  (102) $$\begin{array}{c} h\left(x\right)=\frac{\psi \left[\varphi_{N}\left(F_{X}\left(x\right)\right)\right]}{\left(1-F_{X}\left(x\right)\right)}=\frac{\psi \left[F_{X_{N:N}}\left(x\right)\right]}{{\bar{F}}_{X}\left(x\right)}, \end{array}$$
being nonnegative decreasing. From Lemma 4, (99), and (101), we have for all *t* > 0,
  (103) $$\begin{array}{c} \int_{0}^{t}\psi \left[F_{X_{N:N}}\left(x\right)\right]\left[\frac{f_{X_{N:N}}\left(t\right)}{h_{Z_{N:N}}\left[H_{Z_{N:N}}^{-1}\left(F_{X_{N:N}}\left(t\right)\right)\right]}-\frac{f_{X_{N:N}}\left(x\right)}{h_{Z_{N:N}}\left[H_{Z_{N:N}}^{-1}\left(F_{X_{N:N}}\left(x\right)\right)\right]}\right]\text{d}x\geq 0. \end{array}$$
That is, *X*_*N*:*N*_≤_gdmtfr_*Z*_*N*:*N*_ w.r.t. *ψ*.
(2) Suppose that *X*_*N*:*N*_≤_gdmtfr_*Z*_*N*:*N*_ w.r.t. *ψ*. This asserts that, for all *t* > 0,
  (104) $$\begin{array}{c} \int_{0}^{t}\psi \left[F_{X_{N:N}}\left(x\right)\right]\left[\frac{f_{X_{N:N}}\left(t\right)}{h_{Z_{N:N}}\left[H_{Z_{N:N}}^{-1}\left(F_{X_{N:N}}\left(t\right)\right)\right]}-\frac{f_{X_{N:N}}\left(x\right)}{h_{Z_{N:N}}\left[H_{Z_{N:N}}^{-1}\left(F_{X_{N:N}}\left(x\right)\right)\right]}\right]\text{d}x\geq 0. \end{array}$$
Since the function *ψ*∘*φ*_*N*_(·) is anti-star-shaped with respect to the point (1,0), the function (1 − *u*)/*ψ*[*φ*_*N*_(*u*)] is decreasing in *u* ∈ (0,1), and this leads to the function
  (105) $$\begin{array}{c} h\left(x\right)=\frac{\left(1-F_{X}\left(x\right)\right)}{\psi \left[\varphi_{N}\left(F_{X}\left(x\right)\right)\right]}=\frac{{\bar{F}}_{X}\left(x\right)}{\psi \left[F_{X_{N:N}}\left(x\right)\right]}, \end{array}$$
being nonnegatively decreasing. From Lemma 4, (104), and (101), we have
  (106) $$\begin{array}{c} \int_{0}^{t}{\bar{F}}_{X}\left(x\right)\left[\frac{f_{X}\left(t\right)}{h_{Z}\left[H_{Z}^{-1}\left(F_{X}\left(t\right)\right)\right]}-\frac{f_{X}\left(x\right)}{h_{Z}\left[H_{Z}^{-1}\left(F_{X}\left(x\right)\right)\right]}\right]\text{d}x\geq 0,\;\text{for all }t>0. \end{array}$$
That is, *X*≤_dmtfr_*Y*.
(3) Suppose that *X*≤_dmtfr_*Z*. Then,
  (107) $$\begin{array}{c} \int_{0}^{t}{\bar{F}}_{X}\left(x\right)\left[\frac{f_{X}\left(t\right)}{h_{Z}\left[H_{Z}^{-1}\left(F_{X}\left(t\right)\right)\right]}-\frac{f_{X}\left(x\right)}{h_{Z}\left[H_{Z}^{-1}\left(F_{X}\left(x\right)\right)\right]}\right]\text{d}x\geq 0,\;\text{for all }t>0. \end{array}$$
Shaked and Shanthikumar [8] showed that
  (108) $$\begin{array}{c} H_{Z_{1:N}}^{-1}\left(F_{X_{1:N}}\left(x\right)\right)=H_{Z}^{-1}\left(F_{X}\left(x\right)\right). \end{array}$$
It can be proven that
  (109) $$\begin{array}{c} \frac{f_{X_{1:N}}\left(x\right)}{h_{Z_{1:N}}\left[H_{Z_{1:N}}^{-1}\left(F_{X_{1:N}}\left(x\right)\right)\right]}=\frac{f_{X}\left(x\right)}{h_{Z}\left[H_{Z}^{-1}\left(F_{X}\left(x\right)\right)\right]}. \end{array}$$
Since the function *ψ*(1 − *φ*_*N*_(*u*))/*u* is increasing in *u* ∈ (0,1), the function
  (110) $$\begin{array}{c} h\left(x\right)=\frac{\psi \left[\varphi_{N}\left({\bar{F}}_{X}\left(x\right)\right)\right]}{{\bar{F}}_{X}\left(x\right)}=\frac{\psi \left[F_{X_{1:N}}\left(x\right)\right]}{{\bar{F}}_{X}\left(x\right)}, \end{array}$$
is nonnegative decreasing. From Lemma 4, (107), and (109), we have, for all *t* > 0,
  (111) $$\begin{array}{c} \int_{0}^{t}\psi \left[F_{X_{1:N}}\left(t\right)\right]\left[\frac{f_{X_{1:N}}\left(t\right)}{h_{Z_{1:N}}\left[H_{Z_{1:N}}^{-1}\left(F_{X_{1:N}}\left(t\right)\right)\right]}-\frac{f_{X_{1:N}}\left(x\right)}{h_{Z_{1:N}}\left[H_{Z_{1:N}}^{-1}\left(F_{X_{1:N}}\left(x\right)\right)\right]}\right]\text{d}x\geq 0. \end{array}$$
That is, *X*_1:*N*_≤_gdmtfr_*Z*_1:*N*_ w.r.t. *ψ*.
(4) Suppose that *X*_1:*N*_≤_gdmtfr_*Z*_1:*N*_ w.r.t. *ψ*. Then, for all *t* > 0,
  (112) $$\begin{array}{c} \int_{0}^{t}\psi \left[F_{X_{1:N}}\left(x\right)\right]\left[\frac{f_{X_{1:N}}\left(t\right)}{h_{Z_{1:N}}\left[H_{Z_{1:N}}^{-1}\left(F_{X_{1:N}}\left(t\right)\right)\right]}-\frac{f_{X_{1:N}}\left(x\right)}{h_{Z_{1:N}}\left[H_{Z_{1:N}}^{-1}\left(F_{X_{1:N}}\left(x\right)\right)\right]}\right]\text{d}x\geq 0. \end{array}$$

Since the function *u*/*ψ*(1 − *φ*_*N*_(*u*)) is increasing in *u* ∈ (0,1), the function

(113) $$\begin{array}{c} h\left(x\right)=\frac{{\bar{F}}_{X}\left(x\right)}{\psi \left[\varphi_{N}\left({\bar{F}}_{X}\left(x\right)\right)\right]}=\frac{{\bar{F}}_{X}\left(x\right)}{\psi \left[F_{X_{1:N}}\left(x\right)\right]}, \end{array}$$

is nonnegatively decreasing. From Lemma 4, (112), and (109), we have for all *t* > 0,

(114) $$\begin{array}{c} \int_{0}^{t}{\bar{F}}_{X}\left(x\right)\left[\frac{f_{X_{1:N}}\left(t\right)}{h_{Z_{1:N}}\left[H_{Z_{1:N}}^{-1}\left(F_{X_{1:N}}\left(t\right)\right)\right]}-\frac{f_{X_{1:N}}\left(x\right)}{h_{Z_{1:N}}\left[H_{Z_{1:N}}^{-1}\left(F_{X_{1:N}}\left(x\right)\right)\right]}\right]\text{d}x\geq 0. \end{array}$$

That is, *X*_1:*N*_≤_gdmtfr_*Z*_1:*N*_. Therefore, the proof of the theorem is complete.

**Theorem 12 .**

Assume that *X*≤_gdmtfr_*Z* w.r.t. *ψ*, *ψ*(*u*)=*B*_*n*−*k*+1,*k*_(*u*), *u* ∈ (0,1), for some 1 ≤ *k* ≤ *n*, where *B*_*n*−*k*+1,*k*_ is the distribution function of a beta distribution with parameters *n* − *k*+1 and *k*. Then, we have

(115) $$\begin{array}{c} X_{k:n}\leq_{\text{dmtfr}}Z_{k:n},\;\text{for all }k=1,2,\ldots ,n. \end{array}$$

**Proof**

Suppose that *X*≤_gdmtfr_*Z* w.r.t. *ψ*. This asserts that

(116) $$\begin{array}{c} \int_{0}^{t}\psi \left[F_{X}\left(x\right)\right]\left[\frac{f_{X}\left(t\right)}{h_{Z}\left[H_{Z}^{-1}\left(F_{X}\left(t\right)\right)\right]}-\frac{f_{X}\left(x\right)}{h_{Z}\left[H_{Z}^{-1}\left(F_{X}\left(x\right)\right)\right]}\right]\text{d}x\geq 0,\;\text{for all }t>0. \end{array}$$

By taking *ψ*(*u*)=*B*_*n*−*k*+1,*k*_(*u*), *u* ∈ (0,1), we get that

(117) $$\begin{array}{c} \int_{0}^{t}{\bar{B}}_{n-k+1,k}\left(F_{X}\left(x\right)\right)\left[\frac{f_{X}\left(t\right)}{h_{Z}\left[H_{Z}^{-1}\left(F_{X}\left(t\right)\right)\right]}-\frac{f_{X}\left(x\right)}{h_{Z}\left[H_{Z}^{-1}\left(F_{X}\left(x\right)\right)\right]}\right]\text{d}x\geq 0,\;\text{for all }t>0. \end{array}$$

In view of the fact that

(118) $$\begin{array}{c} B_{n-k+1,k}\left({\bar{F}}_{X}\left(x\right)\right)={\bar{F}}_{X_{k:n}}\left(x\right),\\ \frac{f_{X}\left(x\right)}{h_{Z}\left[H_{Z}^{-1}\left(F_{X}\left(x\right)\right)\right]}=\frac{f_{X_{k:n}}\left(x\right)}{h_{Z_{k:n}}\left[H_{Z_{k:n}}^{-1}\left(F_{X_{k:n}}\left(x\right)\right)\right]}, \end{array}$$

thus

(119) $$\begin{array}{c} \int_{0}^{t}{\bar{F}}_{X_{k:n}}\left(x\right)\left[\frac{f_{X_{k:n}}\left(t\right)}{h_{Z_{k:n}}\left[H_{Z_{k:n}}^{-1}\left(F_{X_{k:n}}\left(t\right)\right)\right]}-\frac{f_{X_{k:n}}\left(x\right)}{h_{Z_{k:n}}\left[H_{Z_{k:n}}^{-1}\left(F_{X_{k:n}}\left(x\right)\right)\right]}\right]\text{d}x\geq 0,\;\text{for all }t>0, \end{array}$$

which is equivalent to

(120) $$\begin{array}{c} X_{k:n}\leq_{\text{dmtfr}}Z_{k:n},\;\text{for all }k=1,2,\ldots ,n. \end{array}$$

Hence, the proof is complete.

Let *k*=1 and *k*=*n* in Theorem 12, respectively, we have the following corollary.

**Corollary 1 .**

Assume that *X*≤_gdmtfr_*Z* w.r.t. *ψ*.

- If *ψ*(*u*)=*B*_*n*,1_(*u*)=∫_0_^*u*^*nx*^*n*−1^d*x*=*u*^*n*^, *u* ∈ (0,1). Then, *X*_1:*n*_≤_dmtfr_*Z*_1:*n*_.
- If *ψ*(*u*)=*B*_1,*n*_(*u*)=∫_0_^*u*^*n*(1 − *x*)^*n*−1^d*x*=1 − (1 − *u*)^*n*^, *u* ∈ (0,1). Then *X*_*n*:*n*_≤_dmtfr_*Z*_*n*:*n*_.

**Theorem 13 .**

Assume that *X*_1_, *X*_2_,…, and *Z*_1_, *Z*_2_,…, are the i.i.d. copies of *X* and *Z*, respectively. Let *N* be a positive integer-valued random variable that is independent of *X*_*i*_'s and *Y*_*i*_'s.

(1) Let the function *ψ* be star-shaped with respect to the point (1,0).
  - If *X*_1:*N*_≤_dmtfr_*Z*_1:*N*_, then *X*_1:*N*_≤_gdmtfr_*Z*_1:*N*_ w.r.t. *ψ*.
  - If *X*_*N*:*N*_≤_dmtfr_*Z*_*N*:*N*_, then *X*_*N*:*N*_≤_gdmtfr_*Z*_*N*:*N*_ w.r.t. *ψ*.
(2) Let the function *ψ* be anti-star-shaped with respect to the point (1,0).
  - If *X*_1:*N*_≤_gdmtfr_*Z*_1:*N*_ w.r.t. *ψ*, then *X*_1:*N*_≤_dmtfr_*Z*_1:*N*_.
  - If *X*_*N*:*N*_≤_gdmtfr_*Z*_*N*:*N*_ w.r.t. *ψ*, then *X*_*N*:*N*_≤_dmtfr_*Z*_*N*:*N*_.

**Proof**

We only give the proof for the case (1) (b), the proofs for other cases are similar and hence omitted. If *X*_*N*:*N*_≤_dmtfr_*Z*_*N*:*N*_, from (30), we obtain that, for all *t* ≥ 0,

(121) $$\begin{array}{c} \int_{0}^{t}{\bar{F}}_{X_{N:N}}\left(x\right)\cdot \left[\frac{f_{X_{N:N}}\left(t\right)}{h_{Z_{N:N}}\left[H_{Z_{N:N}}^{-1}\left(F_{X_{N:N}}\left(t\right)\right)\right]}-\frac{f_{X_{N:N}}\left(x\right)}{h_{Z_{N:N}}\left[H_{Z_{N:N}}^{-1}\left(F_{X_{N:N}}\left(x\right)\right)\right]}\right]\text{d}x\geq 0. \end{array}$$

Since the function *ψ* is star-shaped with respect to the point (1,0), then the function

(122) $$\begin{array}{c} \frac{\psi \left(u\right)}{\left(1-u\right)}, \end{array}$$

is decreasing in *u* ∈ (0,1). Hence, $\psi \left[F_{X_{N:N}}\left(x\right)\right]/{\bar{F}}_{X_{N:N}}\left(x\right)$ is decreasing in *x* ≥ 0; from Lemma 4 and inequality (121), we have for all *t* ≥ 0,

(123) $$\begin{array}{c} \int_{t}^{\infty }\psi \left[F_{X_{N:N}}\left(x\right)\right]\cdot \left[\frac{f_{X_{N:N}}\left(t\right)}{h_{Z_{N:N}}\left[H_{Z_{N:N}}^{-1}\left(F_{X_{N:N}}\left(t\right)\right)\right]}-\frac{f_{X_{N:N}}\left(x\right)}{h_{Z_{N:N}}\left[H_{Z_{N:N}}^{-1}\left(F_{X_{N:N}}\left(x\right)\right)\right]}\right]\text{d}x\geq 0. \end{array}$$

That is, *X*_*N*:*N*_≤_gdmtfr_*Z*_*N*:*N*_ w.r.t. *ψ* as claimed.

**Theorem 14 .**

1. Let the function *ψ* be star-shaped of order *n* with respect to the point (1,0). If *X*_1:*n*_≤_dmtfr_*Z*_1:*n*_, then *X*≤_gdmtfr_*Z* w.r.t. *ψ*.
2. Let the function *ψ* be dual anti-star-shaped of order *n*. If *X*_*n*:*n*_≤_dmtfr_*Z*_*n*:*n*_, then *X*≤_gdmtfr_*Z* w.r.t. *ψ*.

**Proof**

We only give the proof for case (1), the proof for case (2) is similar, and hence omitted. If *X*_1:*n*_≤_dmtfr_*Z*_1:*n*_, from (30), we obtain that

(124) $$\begin{array}{c} \int_{0}^{t}{\bar{F}}_{X_{1:n}}\left(x\right)\cdot \left[\frac{f_{X_{1:n}}\left(t\right)}{h_{Z_{1:n}}\left[H_{Z_{1:n}}^{-1}\left(F_{X_{1:n}}\left(t\right)\right)\right]}-\frac{f_{X_{1:n}}\left(x\right)}{h_{Z_{1:n}}\left[H_{Z_{1:n}}^{-1}\left(F_{X_{1:n}}\left(x\right)\right)\right]}\right]\text{d}x\geq 0,\;\text{for all }t\geq 0. \end{array}$$

Shaked and Shanthikumar [8] showed that *H*_*Z*_*n*:*n*__^−1^(*F*_*X*_*n*:*n*__(*x*))=*H*_*Z*_^−1^(*F*_*X*_(*x*)). It can be proven that

(125) $$\begin{array}{c} \frac{f_{X_{1:n}}\left(x\right)}{h_{Z_{1:n}}\left[H_{Z_{1:n}}^{-1}\left(F_{X_{1:n}}\left(x\right)\right)\right]}=\frac{f_{X}\left(x\right)}{h_{Z}\left[H_{Z}^{-1}\left(F_{X}\left(x\right)\right)\right]},\;\text{for all }x\geq 0. \end{array}$$

Since the function *ψ*(*x*) is star-shaped of order *n* with respect to the point (1,0), then the function *ψ*(*x*)/(1 − *x*)^*n*^ is decreasing in *x* ∈ (0,1). Hence, the function

(126) $$\begin{array}{c} \frac{\psi \left[F_{X}\left(x\right)\right]}{{\left[{\bar{F}}_{X}\left(x\right)\right]}^{n}}, \end{array}$$

is nonnegatively decreasing in *x* ≥ 0. From Lemma 4, inequality (124), and equation (125), we have

(127) $$\begin{array}{c} \int_{0}^{t}\psi \left[F_{X}\left(x\right)\right]\cdot \left[\frac{f_{X}\left(x\right)}{h_{Z}\left[H_{Z}^{-1}\left(F_{X}\left(x\right)\right)\right]}-\frac{f_{X}\left(t\right)}{h_{Z}\left[H_{Z}^{-1}\left(F_{X}\left(t\right)\right)\right]}\right]\text{d}x\geq 0,\;\text{for all }t>0. \end{array}$$

That is, *X*≤_gdmtfr_*Z* w.r.t. *ψ* as claimed.

At the end of this section, we show the relation between the orders DMTFR, RDMTFR, and GDMTFR:

(128) $$\begin{array}{c} X\leq_{\text{gdmtfr}}Zw.r.t.\psi ,\;\psi \left(u\right)=1-u,u\in \left(0,1\right)⟺X\leq_{\text{dmtfr}}Z,\\ X\leq_{\text{gdmtfr}}Zw.r.t.\psi ,\;\psi \left(u\right)=g_{Y}\left[G_{Y}^{-1}\left(u\right)\right],u\in \left(0,1\right)⟺X\leq_{\text{rdmtfr}}Z,\\ X\leq_{\text{rdmtfr}}Z\text{w}.\text{r}.\text{t}.Y,\;Y\text{ is exponential }⟺X\leq_{\text{dmtfr}}Z. \end{array}$$

## 4. Closure and Reversed Closure Properties of the GDMTFR Order

In this section, we investigate the closure and reversed closure properties of the GDMTFR order with respect to the referred functions and the compared random variables, respectively.

The following Theorem 15 indicates that the GDMTFR order has the closure property with respect to the referred function under multiplication of functions.

**Theorem 15 .**

If *X*≤_gdmtfr_*Z* w.r.t. *ψ*, and *φ* is nonnegative decreasing in *x* ∈ (0,1), then

(129) $$\begin{array}{c} X\leq_{\text{gdmtfr}}Zw.r.t.\varphi \cdot \psi . \end{array}$$

**Proof**

Suppose that *X*≤_gdmtfr_*Z* w.r.t. *ψ*. Then, from (30), we have

(130) $$\begin{array}{c} \int_{0}^{t}\psi \left[F_{X}\left(x\right)\right]\left[\frac{f_{X}\left(t\right)}{g_{Y}\left[G_{Y}^{-1}\left(F_{X}\left(t\right)\right)\right]}-\frac{f_{X}\left(x\right)}{g_{Y}\left[G_{Y}^{-1}\left(F_{X}\left(x\right)\right)\right]}\right]\text{d}x\geq 0,\;\text{for all }t>0. \end{array}$$

Since *φ*[*F*_*X*_(*t*)] is nonnegatively decreasing in *x* ∈ (0,1), from (130) and Lemma 4, we obtain for all *t* > 0,

(131) $$\begin{array}{c} \int_{0}^{t}\varphi \left[F_{X}\left(x\right)\right]\cdot \psi \left[F_{X}\left(x\right)\right]\left[\frac{f_{X}\left(t\right)}{g_{Y}\left[G_{Y}^{-1}\left(F_{X}\left(t\right)\right)\right]}-\frac{f_{X}\left(x\right)}{g_{Y}\left[G_{Y}^{-1}\left(F_{X}\left(x\right)\right)\right]}\right]\text{d}x\geq 0, \end{array}$$

which is equivalent to that *X*≤_gdmtfr_*Z* w.r.t. *ψ* · *φ*. Therefore, the proof of the theorem is complete.

From Theorem 15, we have the following corollary.

**Corollary 2 .**

If *X*≤_gdmtfr_*Z* w.r.t. *ψ*, and *ψ* is decreasing in *x* ∈ (0,1), then

(132) $$\begin{array}{c} X\leq_{\text{gdmtfr}}Zw.r.t.\psi^{n}. \end{array}$$

**Theorem 16 .**

Let *ψ*_1_ and *ψ*_2_ be two continuous nonnegative functions defined on (0,1), *X* and *Z* be two continuous nonnegative random variables. Then, the following statements are true.

(1) If *X*≤_gdmtfr_*Z* w.r.t. *ψ*_*i*_, *i*=1,2, then
  (133) $$\begin{array}{c} X\leq_{\text{gdmtfr}}Zw.r.t.\psi_{1}+\psi_{2}. \end{array}$$
(2) If *X*≤_gdmtfr_*Z* w.r.t. *ψ*_1_ and *ψ*_2_ is increasing in *x* ∈ (0,1), then
  (134) $$\begin{array}{c} X\leq_{\text{gdmtfr}}Zw.r.t.\frac{\psi_{1}}{\psi_{2}}. \end{array}$$
(3) If *X*≤_gdmtfr_*Z* w.r.t. *ψ*_2_, *ψ*_1_ is star-shaped and *ψ*_2_ is decreasing, then
  (135) $$\begin{array}{c} X\leq_{\text{gdmtfr}}Zw.r.t.\psi_{1}{}^{\circ}\psi_{2}, \end{array}$$
where *ψ*_1_°*ψ*_2_ is the compound of *ψ*_1_ and *ψ*_2_.
(4) If *X*≤_gdmtfr_*Z* w.r.t. *ψ*_2_, *ψ*_1_ is anti-star-shaped and *ψ*_2_ is increasing, then
  (136) $$\begin{array}{c} X\leq_{\text{gdmtfr}}Zw.r.t.\psi_{1}{}^{\circ}\psi_{2}, \end{array}$$
(5) If *X*≤_gdmtfr_*Z* w.r.t. *ψ*_1_/*ψ*_2_ and *ψ*_2_ is decreasing in *x* ∈ (0,1), then
  (137) $$\begin{array}{c} X\leq_{\text{gdmtfr}}Zw.r.t.\psi_{1}. \end{array}$$
(6) If *X*≤_gdmtfr_*Z* w.r.t. *ψ*_2_ and *ψ*_1_/*ψ*_2_ is decreasing in *x* ∈ (0,1), then
  (138) $$\begin{array}{c} X\leq_{\text{gdmtfr}}Z\text{w.r.t.}\psi_{1}. \end{array}$$

**Proof**

We only give the proofs for the case of (3); the proofs for the other cases are similar and omitted. (3) Suppose that *X*≤_gdmtfr_*Z* w.r.t. *ψ*_2_. Then, from (30), we have

(139) $$\begin{array}{c} \int_{0}^{t}\psi_{2}\left[F_{X}\left(x\right)\right]\left[\frac{f_{X}\left(x\right)}{h_{Z}\left[H_{Z}^{-1}\left(F_{X}\left(x\right)\right)\right]}-\frac{f_{X}\left(t\right)}{h_{Z}\left[H_{Z}^{-1}\left(F_{X}\left(t\right)\right)\right]}\right]\text{d}x\geq 0,\;\text{for all }t>0. \end{array}$$

Since the function *ψ*_2_ is decreasing, *ψ*_2_[*F*_*X*_(*x*)] is then decreasing. Moreover, *ψ*_1_ is star-shaped on the interval (0,1), and then *ψ*_1_(*u*)/*u* is increasing in *u* ∈ (0,1); thus, the function *ψ*_1_[*ψ*_2_(*F*_*X*_(*x*))]/*ψ*_2_[*F*_*X*_(*x*)] is decreasing in *x* ≥ 0. From Lemma 4 and inequality (139), we have for all *t* > 0,

(140) $$\begin{array}{c} \int_{0}^{t}\psi_{1}\left[\psi_{2}\left(F_{X}\left(x\right)\right)\right]\left[\frac{f_{X}\left(x\right)}{h_{Z}\left[H_{Z}^{-1}\left(F_{X}\left(x\right)\right)\right]}-\frac{f_{X}\left(t\right)}{h_{Z}\left[H_{Z}^{-1}\left(F_{X}\left(t\right)\right)\right]}\right]\text{d}x\geq 0, \end{array}$$

which asserts that *X*≤_gdmtfr_*Z* r.t. *ψ*_1_∘*ψ*_2_ as claimed.

Let *X* be a nonnegative absolutely continuous random variable and *ϕ* be a nonnegative increasing function defined on [0, *∞*) with *ϕ*(0)=0. We call that *ϕ* is a generalized scale function and *ϕ*(*X*) is the generalized scale transform of $\dot{X}$. In the accelerated life testing, the *ϕ* is called an accelerating factor.

If a function *ϕ* is increasing convex with *ϕ*(0)=0, then *ϕ* is called a risk preference function, and *ϕ*(*X*) is called the risk preference transform of *X*. If *ϕ* is increasing concave with *ϕ*(0)=0, then *ϕ* is called a risk aversion function, and *ϕ*(*X*) is called the risk aversion transform of *X*. If *ϕ* is a risk aversion function, we say that −*ϕ*^″^/*ϕ*′ is the absolute risk aversion coefficient of *ϕ*. If *ϕ* is a risk preference function, we say that −*ϕ*^″^/*ϕ*′ is the risk preference coefficient of *ϕ*.

**Theorem 17 .**

Let *ψ* be a nonnegative function defined on (0,1) and *ϕ* be a nonnegative function defined on [0, +*∞*) with *ϕ*(0)=0. Suppose that the function *ϕ* is increasing concave with −*ϕ*^″^/*ϕ*′ being decreasing in *x* ≥ 0, and *X*≥_disp_*Z*. Then,

(141) $$\begin{array}{c} X\leq_{\text{gdmtfr}}Z\text{w}.\text{r}.\text{t}.\psi \Rightarrow \phi \left(X\right)\leq_{\text{gdmtfr}}\phi \left(Z\right)w.r.t.\psi . \end{array}$$

**Proof**

Suppose that *X*≤_gdmtfr_*Z* w.r.t. *ψ*. Then, from (30), we have for all *t* > 0,

(142) $$\begin{array}{c} \int_{0}^{t}\psi \left[F_{X}\left(x\right)\right]\left[\frac{f_{X}\left(t\right)}{h_{Z}\left[H_{Z}^{-1}\left(F_{X}\left(t\right)\right)\right]}-\frac{f_{X}\left(x\right)}{h_{Z}\left[H_{Z}^{-1}\left(F_{X}\left(x\right)\right)\right]}\right]\text{d}x\geq 0. \end{array}$$

Since the function *ϕ*(*x*) is increasing concave, *ϕ*′(*x*) is nonnegatively decreasing in *x* ≥ 0. From Lemma 4 and (142), we have

(143) $$\begin{array}{c} \int_{0}^{t}\psi \left[F_{X}\left(x\right)\right]\phi^{\prime }\left(x\right)\left[\frac{f_{X}\left(t\right)}{h_{Z}\left[H_{Z}^{-1}\left(F_{X}\left(t\right)\right)\right]}-\frac{f_{X}\left(x\right)}{h_{Z}\left[H_{Z}^{-1}\left(F_{X}\left(x\right)\right)\right]}\right]\text{d}x\geq 0,\;\text{for all }t>0. \end{array}$$

On the other hand, *ϕ*(*X*)≤_gdmtfr_*ϕ*(*Z*) w.r.t. *ψ* if and only if for all *t* > 0,

(144) $$\begin{array}{c} \int_{0}^{t}\psi \left[F_{\phi \left(X\right)}\left(x\right)\right]\left[\frac{f_{\phi \left(X\right)}\left(t\right)}{h_{\phi \left(Z\right)}\left[H_{\phi \left(Z\right)}^{-1}\left(F_{\phi \left(X\right)}\left(t\right)\right)\right]}-\frac{f_{\phi \left(X\right)}\left(x\right)}{h_{\phi \left(Z\right)}\left[H_{\phi \left(Z\right)}^{-1}\left(F_{\phi \left(X\right)}\left(x\right)\right)\right]}\right]\\ =\int_{0}^{t}\psi \left[F_{X}\left(x\right)\right]\phi^{\prime }\left(x\right)\left[\frac{f_{X}\left(t\right)}{h_{Z}\left[H_{Z}^{-1}\left(F_{X}\left(t\right)\right)\right]}\cdot \frac{\phi^{\prime }\left[\alpha \left(t\right)\right]}{\phi^{\prime }\left(t\right)}-\frac{f_{X}\left(x\right)}{h_{Z}\left[H_{Z}^{-1}\left(F_{X}\left(x\right)\right)\right]}\cdot \frac{\phi^{\prime }\left[\alpha \left(x\right)\right]}{\phi^{\prime }\left(x\right)}\right]\text{d}x\geq 0, \end{array}$$

where *α*(*x*)=*H*_*Z*_^−1^(*F*_*X*_(*x*)). Under the given conditions of the theorem, we have that the function

(145) $$\begin{array}{c} \frac{\phi^{\prime }\left[\alpha \left(x\right)\right]}{\phi^{\prime }\left(x\right)}, \end{array}$$

is increasing and nonnegative for all *x* > 0. Hence, from (143), we obtain for all *t* > 0,

(146) $$\begin{array}{c} \int_{0}^{t}\psi \left[F_{X}\left(x\right)\right]\phi^{\prime }\left(x\right)\left[\frac{f_{X}\left(t\right)}{h_{Z}\left[H_{Z}^{-1}\left(F_{X}\left(t\right)\right)\right]}\cdot \frac{\phi^{\prime }\left[\alpha \left(t\right)\right]}{\phi^{\prime }\left(t\right)}-\frac{f_{X}\left(x\right)}{h_{Z}\left[H_{Z}^{-1}\left(F_{X}\left(x\right)\right)\right]}\cdot \frac{\phi^{\prime }\left[\alpha \left(x\right)\right]}{\phi^{\prime }\left(x\right)}\right]\text{d}x\geq \\ \frac{\phi^{\prime }\left[\alpha \left(t\right)\right]}{\phi^{\prime }\left(t\right)}\int_{0}^{t}\psi \left[F_{X}\left(x\right)\right]\phi^{\prime }\left(x\right)\left[\frac{f_{X}\left(x\right)}{h_{Z}\left[H_{Z}^{-1}\left(F_{X}\left(x\right)\right)\right]}-\frac{f_{X}\left(x\right)}{h_{Z}\left[H_{Z}^{-1}\left(F_{X}\left(t\right)\right)\right]}\right]\text{d}x\geq 0. \end{array}$$

That is to say, inequality (144) is valid, so the proof of the theorem is complete.

**Remark 7 .**

Theorem 17 says, under some appropriate conditions, that the order ≤_gdmtfr_ has the closure property under the concave generalized scale transform.

**Remark 8 .**

Theorem 17 also says, under some appropriate conditions, that the order ≤_gdmtfr_ has the closure property under the action of an increasing concave accelerating factor.

**Remark 9 .**

Theorem 17 also says, under the condition of *X*≥_disp_*Z*, that the GDMTFR order has the closure property under the risk averse transform *ϕ* with an increasing absolute risk aversion coefficient −*ϕ*^″^/*ϕ*′.

With a similar manner of the proof of Theorem 17, Theorem 18 can be proven, and the detailed proof is omitted.

**Theorem 18 .**

Let *ψ* be a nonnegative function defined on (0,1) and *ϕ* be a nonnegative function defined on [0, +*∞*) with *ϕ*(0)=0. Suppose that the function *ϕ* is increasing and convex with *ϕ*^″^/*ϕ*′ being increasing in *x* ≥ 0. If *ϕ*(*X*)≤_gdmtfr_*ϕ*(*Z*) w.r.t. *ψ* and *X*≥_disp_*Z*, then

(147) $$\begin{array}{c} X\leq_{\text{gdmtfr}}Zw.r.t.\psi . \end{array}$$

**Remark 10 .**

Theorem 18 says, under some appropriate conditions, that the order ≤_gdmtfr_ has the reversed closure property under the convex generalized scale transform.

**Remark 11 .**

Theorem 18 also says, under some appropriate conditions, that the order ≤_gdmtfr_ has the reversed closure property under the action of an increasing and convex accelerating factor.

**Remark 12 .**

Theorem 18 also says, under the condition of *X*≥_disp_*Z*, that the GDMTFR order has the reversed closure property under the risk preference transform *ϕ* with an increasing risk preference coefficient *ϕ*^″^/*ϕ*′.

**Theorem 19 .**

Let *ψ* be a nonnegative increasing function defined on (0,1) and let *ϕ* be a nonnegative increasing concave function defined on [0, +*∞*) with *ϕ*(0)=0 and with *ϕ*^″^/*ϕ*′ being decreasing in *x* > 0. Suppose that *X*≥_disp_*Z*. Then,

(148) $$\begin{array}{c} X\leq_{\text{gdmtfr}}Zw.r.t.\psi \Rightarrow \phi \left(X\right)\leq_{\text{gdmtfr}}\phi \left(Z\right)w.r.t.\phi \circ \psi . \end{array}$$

**Proof**

Suppose that *X*≤_gdmtfr_*Z* w.r.t. *ψ*. Then, from (30), we have

(149) $$\begin{array}{c} \int_{0}^{t}\psi \left[F_{X}\left(x\right)\right]\left[\frac{f_{X}\left(t\right)}{h_{Z}\left[H_{Z}^{-1}\left(F_{X}\left(t\right)\right)\right]}-\frac{f_{X}\left(x\right)}{h_{Z}\left[H_{Z}^{-1}\left(F_{X}\left(x\right)\right)\right]}\right]\text{d}x\geq 0,\;\text{for all }t>0. \end{array}$$

Since the function *ϕ*(*x*) is increasing concave, *ϕ*′(*x*) is nonnegatively decreasing in *x* > 0. From Lemma 4 and (149), we have

(150) $$\begin{array}{c} \int_{0}^{t}\psi \left[F_{X}\left(x\right)\right]\phi^{\prime }\left(x\right)\left[\frac{f_{X}\left(t\right)}{h_{Z}\left[H_{Z}^{-1}\left(F_{X}\left(t\right)\right)\right]}-\frac{f_{X}\left(x\right)}{h_{Z}\left[H_{Z}^{-1}\left(F_{X}\left(x\right)\right)\right]}\right]\text{d}x\geq 0,\;\text{for all }t>0. \end{array}$$

Since *ϕ* is nonnegatively increasing concave, *ϕ*(*x*)/*x* is nonnegatively decreasing and *ψ* being nonnegatively increasing leads to increasing *ψ*(*F*_*X*_(*x*)), so *ϕ*[*ψ*(*F*_*X*_(*x*))]/*ψ*(*F*_*X*_(*x*)) is nonnegatively decreasing. From Lemma 4 and (150), we obtain that

(151) $$\begin{array}{c} \int_{0}^{t}\phi \left[\psi \left(F_{X}\left(x\right)\right)\right]\phi^{\prime }\left(x\right)\left[\frac{f_{X}\left(t\right)}{h_{Z}\left[H_{Z}^{-1}\left(F_{X}\left(t\right)\right)\right]}-\frac{f_{X}\left(x\right)}{h_{Z}\left[H_{Z}^{-1}\left(F_{X}\left(x\right)\right)\right]}\right]\text{d}x\geq 0,\;\text{for all t}>\text{0}. \end{array}$$

On the other hand, *ϕ*(*X*)≤_gdmtfr_*ϕ*(*Z*) w.r.t. *ϕ*∘*ψ* if and only if for all *t* ≥ 0,

(152) $$\begin{array}{c} \int_{0}^{t}\phi \left[\psi \left(F_{\phi \left(X\right)}\left(x\right)\right)\right]\left[\frac{f_{\phi \left(X\right)}\left(t\right)}{h_{\phi \left(Z\right)}\left[H_{\phi \left(Z\right)}^{-1}\left(F_{\phi \left(X\right)}\left(t\right)\right)\right]}-\frac{f_{\phi \left(X\right)}\left(x\right)}{h_{\phi \left(Z\right)}\left[H_{\phi \left(Z\right)}^{-1}\left(F_{\phi \left(X\right)}\left(x\right)\right)\right]}\right]\\ =\int_{0}^{t}\phi \left[\psi \left(F_{X}\left(x\right)\right)\right]\phi^{\prime }\left(x\right)\left[\frac{f_{X}\left(t\right)}{h_{Z}\left[H_{Z}^{-1}\left(F_{X}\left(t\right)\right)\right]}\cdot \frac{\phi^{\prime }\left[\alpha \left(t\right)\right]}{\phi^{\prime }\left(t\right)}-\frac{f_{X}\left(x\right)}{h_{Z}\left[H_{Z}^{-1}\left(F_{X}\left(x\right)\right)\right]}\cdot \frac{\phi^{\prime }\left[\alpha \left(x\right)\right]}{\phi^{\prime }\left(x\right)}\right]\text{d}x\geq 0, \end{array}$$

where *α*(*x*)=*H*_*Z*_^−1^(*F*_*X*_(*x*)). Since *ϕ*^″^/*ϕ*′ is decreasing, and *X*≥_disp_*Z*, we have that the function

(153) $$\begin{array}{c} \frac{\phi^{\prime }\left[\alpha \left(x\right)\right]}{\phi^{\prime }\left(x\right)}, \end{array}$$

is nonnegatively increasing in *x* > 0. Therefore, for all *t* > 0,

(154) $$\begin{array}{c} \int_{0}^{t}\phi \left[\psi \left(F_{X}\left(x\right)\right)\right]\phi^{\prime }\left(x\right)\left[\frac{f_{X}\left(t\right)}{h_{Z}\left[H_{Z}^{-1}\left(F_{X}\left(t\right)\right)\right]}\cdot \frac{\phi^{\prime }\left[\alpha \left(t\right)\right]}{\phi^{\prime }\left(t\right)}-\frac{f_{X}\left(x\right)}{h_{Z}\left[H_{Z}^{-1}\left(F_{X}\left(x\right)\right)\right]}\cdot \frac{\phi^{\prime }\left[\alpha \left(x\right)\right]}{\phi^{\prime }\left(x\right)}\right]\text{d}x\\ \geq \frac{\phi^{\prime }\left[\alpha \left(t\right)\right]}{\phi^{\prime }\left(t\right)}\int_{0}^{t}\phi \left[\psi \left(F_{X}\left(x\right)\right)\right]\phi^{\prime }\left(x\right)\left[\frac{f_{X}\left(x\right)}{h_{Z}\left[H_{Z}^{-1}\left(F_{X}\left(x\right)\right)\right]}-\frac{f_{X}\left(x\right)}{h_{Z}\left[H_{Z}^{-1}\left(F_{X}\left(t\right)\right)\right]}\right]\text{d}x\geq 0. \end{array}$$

From (151) and (154), we see that inequality (152) is valid. That is to say, *ϕ*(*X*)≤_gdmtfr_*ϕ*(*Z*) w.r.t. *ϕ*∘*ψ*. Therefore, the proof of the theorem is complete.

**Remark 13 .**

Theorem 19 says that, under some appropriate conditions, the order ≤_gdmtfr_ has the closure property with respect to both the compared random variables and the referred function under the concave generalized scale transforms.

**Remark 14 .**

Theorem 19 also says that, under some appropriate conditions, the order ≤_gdmtfr_ has the closure property with respect to both the compared random variables and the referred function under the action of a concave accelerating factor.

**Remark 15 .**

Theorem 19 also says that, under the condition of *X*≥_disp_*Z*, the GDMTFR order has the closure property with respect to both the compared random variables and the referred function under the risk aversion transform *ϕ* with an increasing absolute risk aversion coefficient −*ϕ*^″^/*ϕ*′.

**Theorem 20 .**

Let *ψ* be a nonnegative increasing function defined on (0,1), and let *ϕ* be a nonnegative increasing convex function defined on [0, +*∞*) with *ϕ*(0)=0 and with −*ϕ*^″^/*ϕ*′ being decreasing in *x* > 0. Assume that *X*≥_disp_*Z*. Then,

(155) $$\begin{array}{c} \phi \left(X\right)\leq_{\text{gdmtfr}}\phi \left(Z\right)w.r.t.\phi \circ \psi \Rightarrow X\leq_{\text{gdmtfr}}Zw.r.t.\psi . \end{array}$$

**Proof**

Suppose that *ϕ*(*X*)≤_gdmtfr_*ϕ*(*Z*) w.r.t. *ϕ*∘*ψ*. Then, from (30), we have, for all *t* > 0,

(156) $$\begin{array}{c} \int_{0}^{t}\phi \left[\psi \left(F_{\phi \left(X\right)}\left(x\right)\right)\right]\left[\frac{f_{\phi \left(X\right)}\left(t\right)}{h_{\phi \left(Z\right)}\left[H_{\phi \left(Z\right)}^{-1}\left(F_{\phi \left(X\right)}\left(t\right)\right)\right]}-\frac{f_{\phi \left(X\right)}\left(x\right)}{h_{\phi \left(Z\right)}\left[H_{\phi \left(Z\right)}^{-1}\left(F_{\phi \left(X\right)}\left(x\right)\right)\right]}\right]\\ =\int_{0}^{t}\phi \left[\psi \left(F_{X}\left(x\right)\right)\right]\phi^{\prime }\left(x\right)\left[\frac{f_{X}\left(t\right)}{h_{Z}\left[H_{Z}^{-1}\left(F_{X}\left(t\right)\right)\right]}\cdot \frac{\phi^{\prime }\left[\alpha \left(t\right)\right]}{\phi^{\prime }\left(t\right)}-\frac{f_{X}\left(x\right)}{h_{Z}\left[H_{Z}^{-1}\left(F_{X}\left(x\right)\right)\right]}\cdot \frac{\phi^{\prime }\left[\alpha \left(x\right)\right]}{\phi^{\prime }\left(x\right)}\right]\text{d}x\geq 0. \end{array}$$

Since *ϕ* is increasing convex, *ϕ*(*y*)/*y* is increasing, and *ψ* is increasing. This leads to *ψ*(*F*_*X*_(*x*)) is nonnegatively increasing. Combining these facts, we obtain that the function

(157) $$\begin{array}{c} \frac{\psi \left(F_{X}\left(x\right)\right)}{\phi \left[\psi \left(F_{X}\left(x\right)\right)\right]}, \end{array}$$

is nonnegative decreasing in *x* > 0. Moreover, since *ϕ* is increasing convex, 1/*ϕ*′ is nonnegatively decreasing. So,

(158) $$\begin{array}{c} \frac{\psi \left(F_{X}\left(x\right)\right)}{\phi \left[\psi \left(F_{X}\left(x\right)\right)\right]\phi^{\prime }\left(x\right)}, \end{array}$$

is nonnegatively decreasing in *x* > 0. From Lemma 4 and (156), we obtain that, for all *t* > 0,

(159) $$\begin{array}{c} \int_{0}^{t}\psi \left(F_{X}\left(x\right)\right)\left[\frac{f_{X}\left(t\right)}{h_{Z}\left[H_{Z}^{-1}\left(F_{X}\left(t\right)\right)\right]}\cdot \frac{\phi^{\prime }\left[\alpha \left(t\right)\right]}{\phi^{\prime }\left(t\right)}-\frac{f_{X}\left(x\right)}{h_{Z}\left[H_{Z}^{-1}\left(F_{X}\left(x\right)\right)\right]}\cdot \frac{\phi^{\prime }\left[\alpha \left(x\right)\right]}{\phi^{\prime }\left(x\right)}\right]\text{d}x\geq 0. \end{array}$$

Under the given conditions that *ϕ* is increasing convex with *ϕ*^″^/*ϕ*′ being decreasing in *x* ≥ 0, and *X*≥_disp_*Z*, we get that *ϕ*′[*α*(*x*)]/*ϕ*′(*x*) is nonnegatively decreasing in *x* > 0. Hence,

(160) $$\begin{array}{c} 0\leq \int_{0}^{t}\psi \left(F_{X}\left(x\right)\right)\left[\frac{f_{X}\left(t\right)}{h_{Z}\left[H_{Z}^{-1}\left(F_{X}\left(t\right)\right)\right]}\cdot \frac{\phi^{\prime }\left[\alpha \left(t\right)\right]}{\phi^{\prime }\left(t\right)}-\frac{f_{X}\left(x\right)}{h_{Z}\left[H_{Z}^{-1}\left(F_{X}\left(x\right)\right)\right]}\cdot \frac{\phi^{\prime }\left[\alpha \left(x\right)\right]}{\phi^{\prime }\left(x\right)}\right]\text{d}x\\ \leq \frac{\phi^{\prime }\left[\alpha \left(t\right)\right]}{\phi^{\prime }\left(t\right)}\int_{0}^{t}\psi \left(F_{X}\left(x\right)\right)\left[\frac{f_{X}\left(t\right)}{h_{Z}\left[H_{Z}^{-1}\left(F_{X}\left(t\right)\right)\right]}-\frac{f_{X}\left(x\right)}{h_{Z}\left[H_{Z}^{-1}\left(F_{X}\left(x\right)\right)\right]}\right]\text{d}x,\;\text{for all }t>0. \end{array}$$

Thus,

(161) $$\begin{array}{c} \int_{0}^{t}\psi \left[F_{X}\left(x\right)\right]\left[\frac{f_{X}\left(t\right)}{h_{Z}\left[H_{Z}^{-1}\left(F_{X}\left(t\right)\right)\right]}-\frac{f_{X}\left(x\right)}{h_{Z}\left[H_{Z}^{-1}\left(F_{X}\left(x\right)\right)\right]}\right]\text{d}x\geq 0. \end{array}$$

That is to say, *ϕ*(*X*)≤_gdmtfr_*ϕ*(*Z*) w.r.t. *ϕ*∘*ψ*. Therefore, the proof of the theorem is complete.

**Remark 16 .**

Theorem 20 says that, under some appropriate conditions, the order ≤_gdmtfr_ has the reversed closure property with respect to both the compared random variables and the referred function under the convex generalized scale transforms.

**Remark 17 .**

Theorem 20 also says that, under some appropriate conditions, the order ≤_gdmtfr_ has the reversed closure property with respect to both the compared random variables and the referred function under the action of a convex accelerating factor.

**Remark 18 .**

Theorem 20 also says that, under the condition of *X*≥_disp_*Z*, the GDMTFR order has the reversed closure property with respect to both the compared random variables and the referred function under the risk preference transform *ϕ* with a decreasing risk preference coefficient *ϕ*^″^/*ϕ*′.

## 5. Examples

We give several illustrative examples that meet the GDMTFR order.

**Example 1 .**

Let *ψ* be a nonnegative function defined on the interval (0,1). Now, we consider comparing two Weibull random variables in the GDMTFR order. Let *X*_*i*_, *i*=1,2, be two Weibull random variables with respective survival function ${\bar{F}}_{X_{i}}$. Specifically, let *X*_*i*_ ~ *W*(*α*_*i*_, *λ*), *i*=1,2, ${\bar{F}}_{X_{i}}\left(x\right)=e^{-\lambda x^{\alpha_{i}}}$, *x* ≥ 0. Hence, the density functions of *X*_*i*_ are, respectively,

(162) $$\begin{array}{c} f_{X_{i}}\left(x\right)=\lambda \alpha_{i}x^{\alpha_{i}-1}e^{-\lambda x^{\alpha_{i}}},\;x\geq 0. \end{array}$$

Then, the GDMTFR order is determined by the parameters *α*_*i*_. Indeed, it is easy to verify that

(163) $$\begin{array}{c} F_{X_{2}}^{-1}\left[F_{X_{1}}\left(x\right)\right]=x^{\alpha_{1}/\alpha_{2}},\\ f_{X_{2}}\left(x\right)=\lambda \alpha_{2}x^{\alpha_{2}-1}e^{-\lambda x^{\alpha_{2}}}. \end{array}$$

Hence,

(164) $$\begin{array}{c} \frac{f_{X_{1}}\left(x\right)}{f_{X_{2}}\left[F_{X_{2}}^{-1}\left(F_{X_{1}}\left(x\right)\right)\right]}=\frac{\alpha_{1}}{\alpha_{2}}x^{\alpha_{1}/\alpha_{2}-1}\text{. } \end{array}$$

1. If *α*_1_ ≥ *α*_2_ > 0, then from (164), it is clear that the function *f*_*X*_1__(*x*)/*f*_*X*_2__[*F*_*X*_2__^−1^(*F*_*X*_1__(*x*))] is increasing in *x* > 0. From this fact and (30), we have that *X*_1_≤_gdmtfr_*X*_2_ w.r.t. *ψ*.
2. If 0 < *α*_1_ ≤ *α*_2_, then from (164), it is clear that the function *f*_*X*_1__(*x*)/*f*_*X*_2__[*F*_*X*_2__^−1^(*F*_*X*_1__(*x*))] is decreasing in *x* > 0. From this fact and (21), we have that *X*_1_≥_gdmtfr_*X*_2_ w.r.t. *ψ*.

**Example 2 .**

Consider a comparison of two beta random variables in the GDMTFR order. Let *ψ* be a nonnegative function defined on the interval (0,1). Let *X*_*i*_, *i*=1,2, be two Beta random variables with respective distribution functions *F*_*X*_*i*__. Specifically, let *X*_*i*_ ~ Beta(*α*_*i*_, 1), *F*_*X*_*i*__(*x*)=*x*^*α*_*i*_^, *x* ∈ (0,1). Hence, the density functions of *X*_*i*_ are given by, respectively,

(165) $$\begin{array}{c} f_{X_{i}}\left(x\right)=\alpha_{i}x^{\alpha_{i}-1},\;x\in \left(0,1\right). \end{array}$$

It is readily verified that

(166) $$\begin{array}{c} F_{X_{2}}^{-1}\left[F_{X_{1}}\left(x\right)\right]=x^{\alpha_{1}/\alpha_{2}},\\ f_{X_{2}}\left(x\right)=\lambda \alpha_{2}x^{\alpha_{2}-1}e^{-\lambda x^{\alpha_{2}}},\;x\in \left(0,1\right). \end{array}$$

Hence,

(167) $$\begin{array}{c} \frac{f_{X_{1}}\left(x\right)}{f_{X_{2}}\left[F_{X_{2}}^{-1}\left(F_{X_{1}}\left(x\right)\right)\right]}=\frac{\alpha_{1}}{\alpha_{2}}x^{\left(\alpha_{1}/\alpha_{2}\right)-1}\text{. } \end{array}$$

1. If *α*_1_ ≥ *α*_2_ > 0, it can be seen that the function *f*_*X*_1__(*x*)/*f*_*X*_2__[*F*_*X*_2__^−1^(*F*_*X*_1__(*x*))] is increasing in *x* ≥ 0. From this fact and (30), we see that *X*_1_≤_gdmtfr_*X*_2_ w.r.t. *ψ*.
2. If 0 < *α*_1_ ≤ *α*_2_, from (171), we see that the function *f*_*X*_1__(*x*)/*f*_*X*_2__[*F*_*X*_2__^−1^(*F*_*X*_1__(*x*))] is decreasing *x* ≥ 0. From this fact and (30), we have that *X*_1_≥_gdmtfr_*X*_2_ w.r.t. *ψ*.

**Example 3 .**

Consider a comparison of another two beta random variables in the GDMTFR order. Let *ψ* be a nonnegative function defined on the interval (0,1). Let *X*_*i*_, *i*=1,2, be two beta random variables with respective distribution function *F*_*X*_*i*__. Specifically, let *X*_*i*_ ~ Beta(1, *β*_*i*_), then the density functions of *X*_*i*_ are given by, respectively,

(168) $$\begin{array}{c} f_{X_{i}}\left(x\right)=\beta_{i}{\left(1-x\right)}^{\beta_{i}-1},x\in \left(0,1\right),\;i=1,2. \end{array}$$

The distribution functions of *X*_*i*_ are thus

(169) $$\begin{array}{c} F_{X_{i}}\left(x\right)=1-{\left(1-x\right)}^{\beta_{i}},x\in \left(0,1\right),\;i=1,2, \end{array}$$

respectively.

It can be verified that

(170) $$\begin{array}{c} F_{X_{2}}^{-1}\left[F_{X_{1}}\left(x\right)\right]=1-{\left(1-x\right)}^{\beta_{1}/\beta_{2}},\\ f_{X_{2}}\left(x\right)=\beta_{2}{\left(1-x\right)}^{\beta_{2}-1},\;x\in \left(0,1\right). \end{array}$$

Hence,

(171) $$\begin{array}{c} \frac{f_{X_{1}}\left(x\right)}{f_{X_{2}}\left[F_{X_{2}}^{-1}\left(F_{X_{1}}\left(x\right)\right)\right]}=\frac{\beta_{1}}{\beta_{2}}{\left(1-x\right)}^{\left(\beta_{1}/\beta_{2}\right)-1}\text{.} \end{array}$$

1. If *β*_1_ ≥ *β*_2_ > 0, it is clear that the function *f*_*X*_1__(*x*)/*f*_*X*_2__[*F*_*X*_2__^−1^(*F*_*X*_1__(*x*))] is decreasing *x* > 0. From this fact and (30), we see that *X*_1_≥_gdmtfr_*X*_2_ w.r.t. *ψ*.
2. Assume that 0 < *β*_1_ ≤ *β*_2_. From (171), we see that *f*_*X*_1__(*x*)/*f*_*X*_2__[*F*_*X*_2__^−1^(*F*_*X*_1__(*x*))] is increasing *x* > 0. From this fact and (30), we see that *X*_1_≤_gdmtfr_*X*_2_ w.r.t. *ψ*.

**Example 4 .**

Let *ψ* be a nonnegative function defined on the interval (0,1); we consider to compare two Pareto random variables in the GDMTFR order. Specifically, assume that

(172) $$\begin{array}{c} X∼\mathbb{P}\left(\alpha_{1},\lambda \right),\;Y∼\mathbb{P}\left(\alpha_{2},\lambda \right), \end{array}$$

where *α*_1_, *α*_2_, *λ* are positive real numbers. That is, for all *x* ≥ 0,

(173) $$\begin{array}{c} {\bar{F}}_{X}\left(x\right)={\left(\frac{\lambda }{\lambda +x}\right)}^{\alpha_{1}},\\ {\bar{G}}_{Y}\left(x\right)={\left(\frac{\lambda }{\lambda +x}\right)}^{\alpha_{2}}\text{,} \end{array}$$

 

(174) $$\begin{array}{c} f_{X}\left(x\right)=\frac{\alpha_{1}}{\lambda }{\left(\frac{\lambda }{\lambda +x}\right)}^{\alpha_{1}+1},\\ g_{Y}\left(x\right)=\frac{\alpha_{2}}{\lambda }{\left(\frac{\lambda }{\lambda +x}\right)}^{\alpha_{2}+1}. \end{array}$$

Then,

(175) $$\begin{array}{c} G_{Y}^{-1}\left(F_{X}\left(x\right)\right)={\bar{G}}_{Y}^{-1}\left({\bar{F}}_{X}\left(x\right)\right)=\lambda {\left(1+\frac{x}{\lambda }\right)}^{\alpha_{1}/\alpha_{2}}-\lambda . \end{array}$$

Hence,

(176) $$\begin{array}{c} \frac{f_{X}\left(x\right)}{g_{Y}\left[G_{Y}^{-1}\left(F_{X}\left(x\right)\right)\right]}=\frac{\alpha_{1}}{\alpha_{2}}{\left(1+\frac{x}{\lambda }\right)}^{\left(\alpha_{1}/\alpha_{2}\right)-1}. \end{array}$$

1. If *α*_1_ ≥ *α*_2_ > 0, then from (176), we see that the function *f*_*X*_(*x*)/*g*_*Y*_[*G*_*Y*_^−1^(*F*_*X*_(*x*))] is increasing in *x* > 0; hence, from (30), we have *X*≤_gdmtfr_*Y* w.r.t. *ψ*.
2. If 0 < *α*_1_ ≤ *α*_2_, then *f*_*X*_(*x*)/*g*_*Y*_[*G*_*Y*_^−1^(*F*_*X*_(*x*))] is decreasing in *x* > 0; from (30), we get that *X*≥_gdmtfr_*Y* w.r.t. *ψ*.

## Data Availability

In the examples of this research, we mainly use random variables to verify the correctness of the theory.
